# Th17/Treg-Related Transcriptional Factor Expression and Cytokine Profile in Patients With Rheumatoid Arthritis

**DOI:** 10.3389/fimmu.2020.572858

**Published:** 2020-12-11

**Authors:** Agnieszka Paradowska-Gorycka, Anna Wajda, Katarzyna Romanowska-Próchnicka, Ewa Walczuk, Ewa Kuca-Warnawin, Tomasz Kmiolek, Barbara Stypinska, Ewa Rzeszotarska, Dominik Majewski, Pawel Piotr Jagodzinski, Andrzej Pawlik

**Affiliations:** ^1^ Department of Molecular Biology, National Institute of Geriatrics, Rheumatology and Rehabilitation, Warsaw, Poland; ^2^ Department of Connective Tissue Diseases, National Institute of Geriatrics, Rheumatology and Rehabilitation, Warsaw, Poland; ^3^ Department of Pathophysiology, Warsaw Medical University, Warsaw, Poland; ^4^ Department of Pathophysiology and Immunology, National Institute of Geriatrics, Rheumatology and Rehabilitation, Warsaw, Poland; ^5^ Department of Rheumatology and Internal Medicine, Poznan University of Medical Science, Poznan, Poland; ^6^ Department of Biochemistry and Molecular Biology, Poznan University of Medical Sciences, Poznan, Poland; ^7^ Department of Physiology, Pomeranian Medical University, Szczecin, Poland

**Keywords:** rheumatoid arthritis, Th17, Treg, transcriptional factor, gene expression, biomarkers, pathogenesis

## Abstract

**Objectives:**

The aim of our study was to determine whether there is a correlation between transcription factors expression and Th17/Treg ratio, cytokine profile in the RA phenotype as well as to identify transcription factors that could be a potential biomarker for RA.

**Methods:**

The study was conducted on 45 patients with RA, 27 patients with OA and 46 healthy controls (HCs). Th17 and Treg frequency was determined by flow cytometry (15 patients with RA/OA and 15 subjects of HC). Gene expression was estimated by qPCR, and the serum cytokine levels were determined by ELISA.

**Results:**

The percentage of Treg (CD4+CD25highCD127-) cells in RA patients was lower than in OA patients or HCs. Proportions of Th17 (CD4+CCR6+CXCR3-) cells were higher in RA and OA in comparison to HCs. *STAT5* showed a very high expression in the blood of RA patients compared to healthy subjects. The expression of *STAT5* and *HELIOS* was not detected in Th17 cells. A positive correlation between *SMAD3* and *STAT3* in RA patients was observed. Negative correlations between *HIF-1A* and *SMAD2* in RA Treg cells and DAS-28 score were observed. The range of serum of IL-17 and IL-21 were higher in RA patients than in OA patients. Concentrations of serum IL-2 and IFN-γ were higher in RA and OA patients than in healthy subjects. Based on the ROC analysis, the diagnostic potential of the combination of *HIF1A*, *SMAD3* and *STAT3*, was determined at AUC 0.95 for distinguishing RA patients from HCs. For distinguishing RA patients from OA patients the diagnostic potential of the combination of *SMAD2*, *SMAD3*, *SMAD4* and *STAT3*, was determined at AUC 0.95.

**Conclusion:**

Based on our study, we conclude that *SMAD3* and *STAT3* could be potential diagnostic biomarkers for RA.

## Introduction

Rheumatoid arthritis (RA) is a chronic autoimmune disease of multifactorial origin (1). Although the pathogenesis of RA is incompletely understood, today we know that induction and progression of the disease is characterized by an abnormal regulatory T cell (Treg) response with a shift towards a Th17 cell response (2, 3). However, the mechanism behind this phenomenon remains unclear. Moreover, the connection of the Th17/Treg cells imbalance with pro-/anti-inflammatory cytokines production is relevant for the development and/or progression of the disease, which is in turn associated with the autoimmunity, chronic inflammation and articular destruction in the joints of RA patients (4, 5).

Th17 cells and Treg cells, each with specific functions and gene expression, develop from the same naive CD4+ T cells, but under different cytokine environment (3). Typical pro-inflammatory Th17 cells, through the induction of proinflammatory cytokines, lead to autoimmune-derived tissue inflammation and joint damage. Furthermore, the activity of Th17 cells, as well as other effector T cells, is suppressed by Treg cells. Treg cells through the production of anti-inflammatory cytokines, are immunosuppressive, maintenance self-tolerance and inhibit autoimmunity. Th17 and Treg cells are characterized by the selective expression of distinct transcriptional factors. *RORc* (retinoic acid receptor-related orphan receptor variant 2) is characteristic for Th17 cells, whereas *FOXP3* (forkhead transcription factor) for Treg cells (6–8). IL-6, IL-21, and IL-23 activate signal transducers and activators of transcription (*STAT*3), which is critical for Th17 cell differentiation. In contrast, IL-2–*STAT5* pathway has been demonstrated to block Th17 differentiation (7, 9, 10). TGF-β alone leads to the activation of the *SMAD2* (Sma- and Mad-related protein2) and *SMAD3* (Sma- and Mad-related protein 3), which are essential for the activity of the *FOXP3* gene and *FOXP3* histone acetylation (11, 12). Moreover, activation of the IL-2–*STAT5* pathway by transcriptional factor *HELIOS* (Ikaros family zinc-finger protein 2; IKZF2) ensures Treg survival and stability (13, 14). Th17/Treg balance is also modulated by hypoxia-inducible factor 1α (*HIF-1A*) and suppressor of cytokine signaling (*SOCS*) proteins. *HIF-1A* promotes Th17 differentiation by induction of RORc2 and in consequence, activates Th17 signature genes, but on the other hand, it inhibits Treg differentiation by *FOXP3* protein degradation. *SOCS1* by regulating the IL-2–mediated STAT5 signaling pathway negatively regulates the Treg number, but on the other hand, *SOCS1* inhibits loss of *FOXP3* and conversion of Tregs to Th1 cells or Th17 cells.

Since a decrease in Treg cells and an increase in the Th17 cells drives the expansion of autoimmunity in patients with RA, establishing the molecular mechanism of Th17/Treg-related transcriptional factors as well as cytokines regulation is essential for understanding RA development and progression. Our study aimed to determine whether there is a correlation between expression of transcription factors and Th17/Treg ratio, cytokine profile in the RA phenotype as well as to identify transcription factors that could be potential diagnostic biomarkers for RA.

## Patients and Methods

### Study Population

The study was conducted on a group of 45 RA patients, 27 osteoarthritis (OA) patients and 46 healthy controls (HCs). Gene expression in blood and cytokine profile was estimated for 45 RA patients, 27 OA patients and 46 healthy controls. Furthermore, the gene expression in Th17/Treg cells was estimated for 15 RA patients, 15 OA patients and 15 healthy subjects. This study meets all criteria contained in the Declaration of Helsinki and was approved by the Ethics Committee of the National Institute of Geriatrics, Rheumatology, and Rehabilitation, Warsaw, Poland (approval protocol number 29 June 2016). All participants gave their written informed consent before enrolment. Patients with RA and OA were recruited from the National Institute of Geriatrics, Rheumatology and Rehabilitation in Warsaw, Poland. All RA patients fulfilled the American College of Rheumatology (ACR 2010) criteria for RA. Patients with OA were diagnosed based on characteristic x-ray findings and the absence of features suggestive of inflammatory arthritis and must meet the ACR criteria for OA of the knee. RA and OA patients with an active infection, cancer or other rheumatological diseases were excluded from the study. The control groups consisted of healthy volunteers who do not show any clinical or laboratory signs of autoimmune diseases. They were randomly selected from blood bank donors to match the patients in age, gender, and ethnicity. Patients and control subjects had the same socioeconomic status and were from the same geographical area.

Patients eligible for the study were evaluated based on physical examination and laboratory tests. Age, gender, disease duration, tender and swollen joints number, C-reactive protein (CRP), erythrocyte sedimentation ratio (ESR), platelets (PLT) and creatinine, presence of rheumatoid factor (RF ≥34 IU/ml), presence of anti-CCP antibodies (anti-cyclic citrullinated peptide autoantibodies, aCCP ≥17 U/ml), disease activity score in 28 joints (DAS-28), visual analog scale (VAS, range 0–100), Larsen score, and the information about the treatment were collected at the time of the clinical materials sampling. Demographic and clinical characteristic of patients is summarized in **Table 1**.

**Table 1:** Demographic and clinical characteristic of the study population.

Parameter	RA, n = 45	OA, n = 27	HC, n = 40
Age, years (median, range)	61 (21–77)	67 (28–85)	51 (41–63)
Female/male	37/8	19/8	32/8
ESR, mm/h (mean ± SD)	43 ± 35	18 ± 11	
CRP, mg/dl (mean ± SD)	23 ± 5	8 ± 6	
Disease duration, years (median, range)	10 (0.5–30)		
DAS-28 (mean ± SD)	5.16 ± 1.38		
VAS (mm)	61.68 ± 22.66		
Larsen	2.75 ± 1.28		
DAS-28 >5,1; n (%)	23 (58%)		
RF positivity, n (%)	28 (68%)		
anti-CCP positivity, n (%)	33 (80%)		
**Medication**			
Methotrexate (MTX), n (%)	24 (53%)		
Leflunomid, n (%)	3 (6%)		
Glucocorticoids, n (%)	18 (40%)		
Antimalarials drug, n (%)	10 (88%)		
Anti-TNF therapy, n (%)	7 (16%)		
Other biologic drug, n (%)	2 (4%)		

### Detection of Th17 and Treg Cells by Flow Cytometry

Peripheral blood mononuclear cells (PBMCs) were isolated by density gradient centrifugation using Ficoll-Paque (GE Healthcare Bio-Sciences, Uppsala, Sweden). Cells were cultured in RPMI 1640 medium (Invitrogen, Paisley, UK), 10% heat-inactivated fetal bovine serum (FBS) (Gibco, Thermofisher, USA), 100 U/ml penicillin and100 μg/ml streptomycin (Sigma-Aldrich) for 12 h. The cells were then harvested and stained for respective membrane antigens using anti-CD4 APC-Cy7, anti-CD25 PE, anti-CD127 FITC, anti-CCR6 APC, and anti-CXCR3 PE-Cy7 murine Abs. After the washing step, cells were acquired, analyzed and sorted using a FACSAria cell sorter/cytometer and Diva software. Dead cells were excluded from analysis by 7AAD staining. All reagents used in flow cytometry analysis were purchased from Becton Dickinson (San Jose, CA, USA). Gating strategy is presented in **Figure 1S in Supplementary Files**.

### RNA Isolation from Whole Blood

Total RNA was extracted from the whole blood using AA Biotech MicroRNA Concentrator (A&A Biotechnology, Poland) with Trizol Reagent (Invitrogen). The quantity and quality of isolated RNA were evaluated by Quawell Q5000 spectrophotometer. cDNA was prepared using High Capacity cDNA Reverse Transcription with RNase Inhibitor Kit (Applied Biosystems, Foster City, CA), according to the manufacturer’s instruction.

### RNA Isolation from Th17 and Treg Cells

Total RNA was extracted from the Th17 and Treg cells using miRNeasy Micro Kit (Qiagen, Germany). The quantity and quality of isolated RNA were evaluated by Quawell Q5000 spectrophotometer. Total RNA obtained during isolation was used to perform the reverse transcription reaction. A commercially available High Capacity cDNA Reverse Transcription Kit (Applied Biosystems, Carlsbad, CA, USA) was used for this purpose according to the manufacturer’s instructions. The synthesized cDNA was stored at −30°C. To increase the sensitivity of further analysis, pre-amplification of cDNA originating from reverse transcription reaction was done using TaqMan^®^ PreAmp Master Mix Kit (Applied Biosystem) according to the manufacturer’s instructions.

### Gene Expression

For analysis of the transcription factors expression following TaqMan primer and probes (Assay; Applied Biosystems, Foster City, CA, USA): *FOXP3* (Hs01085834_m1), *RORc2* (Hs01076112_m1), *SMAD2* (Hs00998187_m1), *SMAD3* (Hs00969210_m1), *SMAD4* (Hs00929647_m1), *STAT3* (Hs00374280_m1), *STAT5a* (Hs00559647_m1), *HIF-1A* (Hs00153153_m1), *SOCS1* (Hs00705164_m1), *HELIOS* (Hs00212361_m1), *GAPDH* (Hs02786624_g1), and *RPLO* (Hs99999902_m1) were used. All assays were guaranteed for their PCR efficiency of 100 ± 2%. For one probe we used: 5ul of the TaqMan Gene Expression Mix (Applied Biosystems, Foster City, CA, USA), 0,5ul Assay, 2ul cDNA and 2,5ul H_2_O. The real-time PCR condition was Hold Stage: 50°C for 2 min and 95°C for 10 min, and PCR Stage: 40 cycles at 95°C for 15 s and 60°C for 1 min. Each sample was analyzed in duplicates and mean Ct value was taken for further analysis. Ct value higher than 35 was taken as below quantification. The most relevant housekeeping gene has been selected and the relative expression was calculated by ΔΔ Ct method or ΔCt method (normalized to *GAPDH* and *RPLO* as reference gene in the case of analysis in whole blood, in the case of sorted Th17 and Treg cells- *RPLO*) using Quant Studio 5 real-time PCR System (Applied Biosystems, Foster City, CA, USA).

### Detection of Protein Levels

For quantitative determination of cytokine serum levels, the samples from RA and OA patients and healthy subjects were separated from peripheral venous blood at room temperature and stored at −86°C until analysis. Levels of Il-17A (pg/ml), IL-17F (pg/ml), IL-10 (pg/ml), TGF-β (pg/ml), IL-23 (pg/ml), IL-21 (pg/ml), IL-22 (pg/ml), IFN-γ (pg/ml), IL-35 (pg/ml), IL-6 (pg/ml) were determined using commercially available enzyme-linked immunosorbent assay (ELISA) kits according to the manufacturer’s instructions (Fine Test, Wuhan, China). The optical density was measured at 450 nm with an automatic ELISA reader (LT-4000 Microplate Reader, Labtech).

### Statistical Analysis

Data were analyzed using GraphPad Prism software version 8.4.2. The Shapiro–Wilk test was used as a normality test. Statistical significance between relative gene expression adjusted by age in RA patients or OA patients in comparison to healthy blood donor was determined using the logistic regression. Patients classification was considered as a general binary classification and a predictor vector (gene expression and age) and response variable y (corresponding group HC or RA/OA). Differences in basal gene expression from sorted cells and protein levels between study groups were determined using non-parametric Kruskal-Wallis test and Dunn’s multiple comparison test or Mann-Whitney U test. Differences between cell populations in groups of patients (HC vs OA vs RA) were analyzed by the ordinary one-way ANOVA with multiple comparisons (when data were normally distributed) or Kruskal-Wallis test with multiple comparisons (when data were not normally distributed). For all tests, a value of p < 0.05 was considered significant. Correlation analysis has been conducted using Spearman or Pearson test. The receiver operating characteristic (ROC) curves analysis, the area under curves, the likelihood ratio chi-square, and p-value obtained by multivariable logistic regression analysis were calculated.

## Results

### Frequencies of Th17 and Treg Cells in Peripheral Blood in Rheumatoid Arthritis Patients, Osteoarthritis Patients, and Healthy Subjects

**Figure 1:**
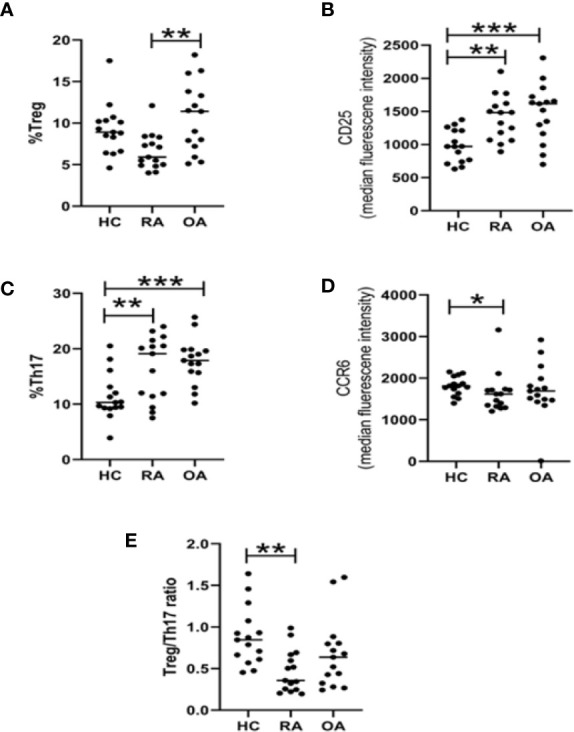
Flow cytometry analysis of Treg and Th17 lymphocyte subpopulations in HC (n = 15), RA (n = 15), and OA (n = 15) blood. Percentage of Tregs cells among CD4 cells **(A)**. Expression of CD25 on Tregs **(B)**. Percentage of Th17 cells among CD4 cells **(C)**. Expression of CCR6 on Th17 **(D)**. Treg/Th17 ratio **(E)**. *p < 0.05, **p < 0.005, ***p < 0.0005.

At first, the Th17 and Treg cells frequencies were determined in PBMCs using flow cytometry (**Figure 1S in Supplementary Files**). For this part of our study, we used blood from 15 RA patients, 15 OA patients and 15 healthy subjects. The percentage of Treg (CD4+CD25highCD127-) cells in RA patients (5, 9%; 4–12, 10%) were lower than in OA patients (11, 40%; 5, 10–18, 20%) or blood of HCs (8, 9%; 4,60–17, 50%). However, the difference was statistically significant only for RA vs OA comparison (**Figure 1A**). We observed that the expression of CD25 on Treg obtained from both RA and OA patients were statistically significantly higher than in HC persons (**Figure 1B**). Proportions of Th17 (CD4+CCR6+CXCR3-) cells were significantly higher in RA (19, 1%; 11, 4–24%) and OA (17, 9%; 10; 2–25, 7%) in comparison to HCs (10, 3%; 3, 9–20, 5%) (**Figure 1C**). On the other hand, Th17 RA was characterized by lower expression of CCR6 than Th17 HC (**Figure 1D**). We created also the Treg/Th17 ratio index to assess balance between anti-inflammatory and proinflammatory subsets of CD4 cells in blood. We found that Treg/Th17 ratio index in RA patients was lower than in HC clearly indicating disturbance in Th response (**Figure 1E**). In RA patients, Th17 cells were five to six times more frequent than Treg cells, while in OA patients and healthy subjects Th17 cells were 1.5 to 2 times more frequent than Treg cells.

### Transcriptional Factors Expression in Whole Blood and Th17/Treg Cells

As the Th17/Treg balance is regulated by different transcriptional factors, in this study we determined the transcriptional factor mRNA level in whole blood and both FACS-sorted Th17 and Treg cells using qPCR.

**Figure 2:**
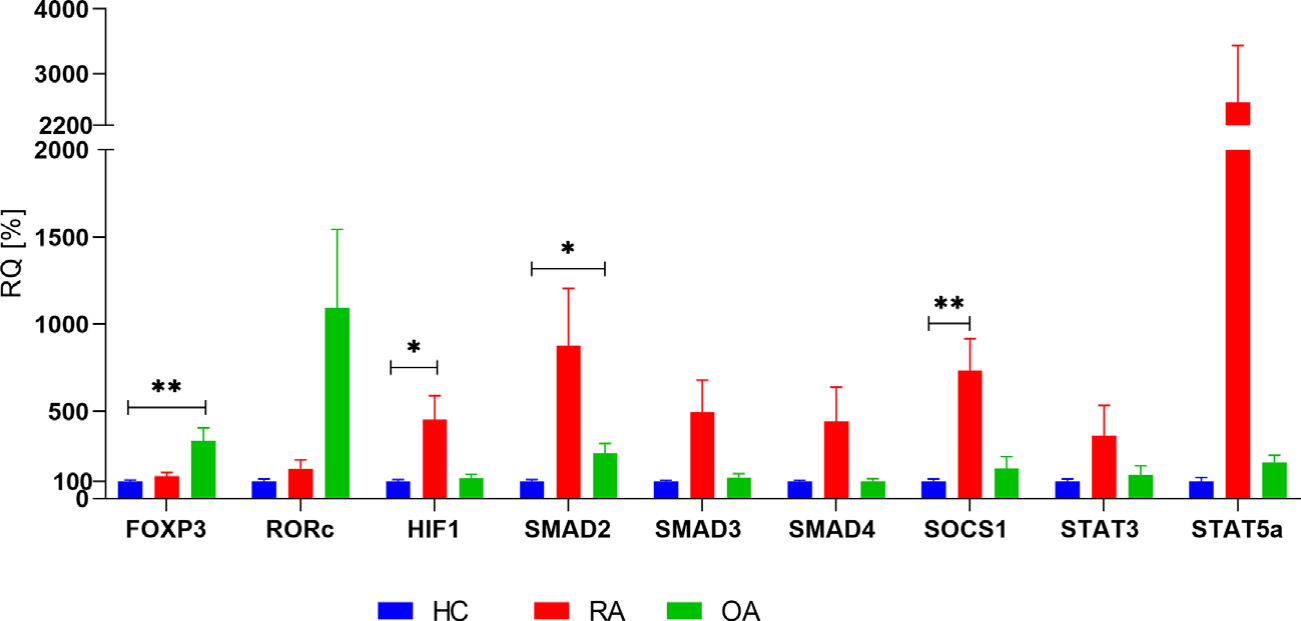
Basal expression of analyzed genes in whole blood in RA, OA and HC (HC relative expression is taken as 100%). Data are presented as mean ± SEM. Significance at p < 0.05 (comparison RA and OA to HC, adjusted by age). *p < 0.05; **p < 0.01.

First, we investigated the mRNA levels of *FOXP3*, *RORc*, *SMAD2*/*3/4*, *SOCS1*, *HIF-1A*, *STAT3*, and *STAT5* in whole blood of patients with RA, OA as well as in healthy controls (HCs). Presented results are adjusted by age, because analyzed groups were significantly different in the aspect of this parameter. We observed that RA patients were characterized by significantly higher level of *HIF-1A* (p = 0.0468), *SOCS1* (p = 0.0065) when compared with healthy subjects. Difference between *SMAD3* in RA and HC was at the border of statistical significance (p = 0.051). *STAT3* (p = 0.38) and *STAT5* (p = 0.069) did not differ when compared expression in RA patients with healthy subjects (**Figure 2**). In the case of *STAT5*, very high range of expression was noted. Furthermore, OA patients were characterized by higher level of *SMAD2* (p = 0.013), *STAT5* (p = 0.0505), *FOXP3* (p = 0.0018) when compared with healthy donors. In the present study all of the analyzed genes, excluding *FOXP3* and *RORc*, revealed higher mRNA expression levels in RA patients in comparison to OA patients. The expression of *HIF-1A*, *SMAD3, SMAD4*, and *STAT5* in whole blood was higher in RA patients than in OA patients. The results of *FOXP3* and *RORc* mRNA expression have shown an inverse trend; patients with RA revealed lower *FOXP3* and *RORc* mRNA expression in comparison with OA patients (p = 0.001 and p= 0.02, respectively). Because in our analyzed groups, age was significantly different, we have checked the difference between gene expression in 1) RA patients in age < 60 vs ≥60; 2) HC vs RA in age <60 and 3) RA vs OA in age >60. Between RA patients (< 60 vs ≥60), we did not observe significant changes. Comparison HC and RA aged to 60 years old (data not shown) revealed significantly higher mRNA *HIF-1A* level (p = 0.02) and *SOCS1* level (p = 0.001) in healthy subjects, whereas *STAT3* expression was higher in RA patients (p = 0.0031). When compared patients over 60 years old who suffered from RA and OA, we did not detect any significant difference in genes expression.

**Figure 3:**
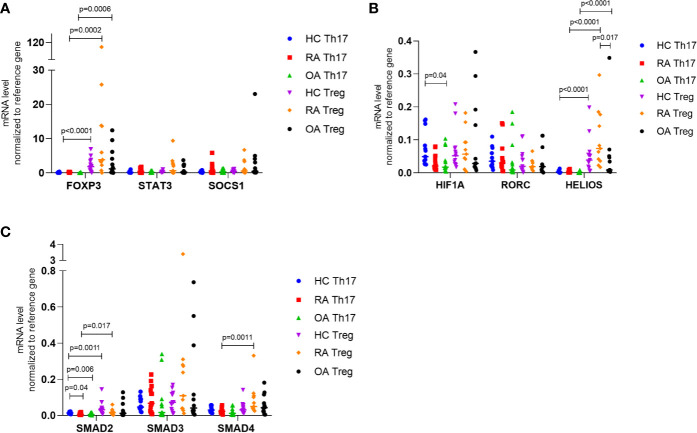
**(A)** *FOXP3, STAT3, SOCS1*; **(B)** *HIF-1A, RORC, HELIOS*; **(C)** *SMAD2, SMAD3, SMAD4* expression in FACS-sorted Th17 and Treg cells in RA, OA and HC normalized to housekeeping gene (RPLO). Data were presented as individual points with median. Significance at p < 0.05 (multiple comparison Kruskal-Wallis test with Dunn’s post hoc between HC, RA and OA; Mann-Whitney test between Th17 and Treg cells).

Next, we investigated the mRNA levels of all genes in Th17 and Treg cells in patients with RA, OA as well as in healthy controls (**Figure 3**). We demonstrated that in all study groups (RA, OA, HCs) the expression of *STAT5* and *HELIOS* was not detected in Th17 cells and that *STAT5* mRNA expression in Treg cells was at a very low level. *HIF-1A* expression was significantly higher in Th17 cells from HC than in Th17 cells from OA patients (p = 0.04). Although *SMAD2* expression was at a low level in Th17 cells, we noticed that *SMAD2* expression was significantly higher in the healthy subjects compared to RA or OA patients (p = 0.04 and p = 0.006, respectively). In comparison between Th17 and Treg cells, the level of *SMAD2* expression was significantly higher in Treg cells from HC (p = 0.0011) and from RA patients (p = 0.017) than in Th17 cells. In RA patients *SMAD4* mRNA level was elevated in Treg cells compared with Th17 cells (p = 0.0011). In comparison between Th17 and Treg cells, the level of *FOXP3* expression was significantly higher in Treg cells in all analyzed groups (in HC p<0.0001, in RA p = 0.0002, in OA p = 0.0006) than in Th17 cells. We also observed that *HELIOS* mRNA level was significantly higher in Treg cells from RA patients compared to Treg cells from OA patients (p = 0.017).

### Correlation Between Transcriptional Factors Expression in Whole Blood As Well As in Th17/Treg Cells

In the next step, we investigated a correlation analysis to indicate a potential direction for further functional research on the mechanisms of the regulation and cell differentiation.

Data showed that in healthy subjects, in whole blood, very strong correlations between *FOXP3* and *SMAD3* (r = 0.7, p<0.0001) and between *SMAD3* and *STAT5a* (r = 0.6; p = 0.001; **Figure 2S in Supplementary Files**) were observed. The expression of *SMAD3* was positively correlated with *STAT3* (r = 0.99, p < 0.0001, **Figure 3S in Supplementary Files**) in Treg cells from healthy subjects. In Th17 cells from healthy subjects, we revealed a very high positive correlation between the expression of *STAT3* and *SMAD3/RORc* (r = 0.94, p < 0.001, r = 0.91, p = 0.0001, respectively; **Figure 3S in Supplementary Files**).

In whole blood of RA patients but not OA patients (**Figure 2S in Supplementary Files**), we noted a very high correlation between *SMAD2* and *SMAD4* (r = 0.7, p < 0.0001).

In RA Treg cells (**Figure 4S in Supplementary Files A–D**) we observed a positive correlation between *SMAD3* and *STAT3* (r = 0.89, p = 0.001) as well as between *HELIOS* and *SMAD4* (r = 0.86, p = 0.001). Whereas, in OA Treg cells, correlation between *HELIOS* and *SMAD4* did not occur.

Furthermore, in RA Th17 cells (**Figure 4S in Supplementary Files E–G**) the significant correlation has been observed between expression of *SMAD3* and *STAT3/RORc/FOXP3* (r = 0.92, p < 0.0001, r = 0.92, p < 0.0001, r = 0.90, p < 0.0001, respectively), expression of *FOXP3* and *STAT3* (r = 0.91, p < 0.0001), expression of *SOCS1* and *STAT3* r = 0.94, p < 0.0001). In OA Th17 cells (**Figure 4S in Supplementary Files M–P**), the highest correlation has been revealed between expression of *SOCS1* and *STAT3* (r = 0.90, p < 0.0001), expression of *SMAD3* and *RORc* (r = 0.89, p < 0.0001), and expression of *SMAD4* and *SMAD3* (r = 0.95, p < 0.0001).

### SOCS1 and STAT3 Downregulation in Treg Cells and Upregulation in Th17 Cells in Rheumatoid Arthritis Patients With DAS28 > 5.1

Considering the importance of the Th17/Treg-related transcriptional factors in the pathogenesis of autoimmune diseases, we also analyzed whether examined Th17/Treg-related transcriptional factors mRNA expression may have an impact on the RA phenotype. Both, transcriptional factors expression in whole blood as well as in Th17/Treg cells was examined concerning clinical parameters of RA patients.

**Figure 4:**
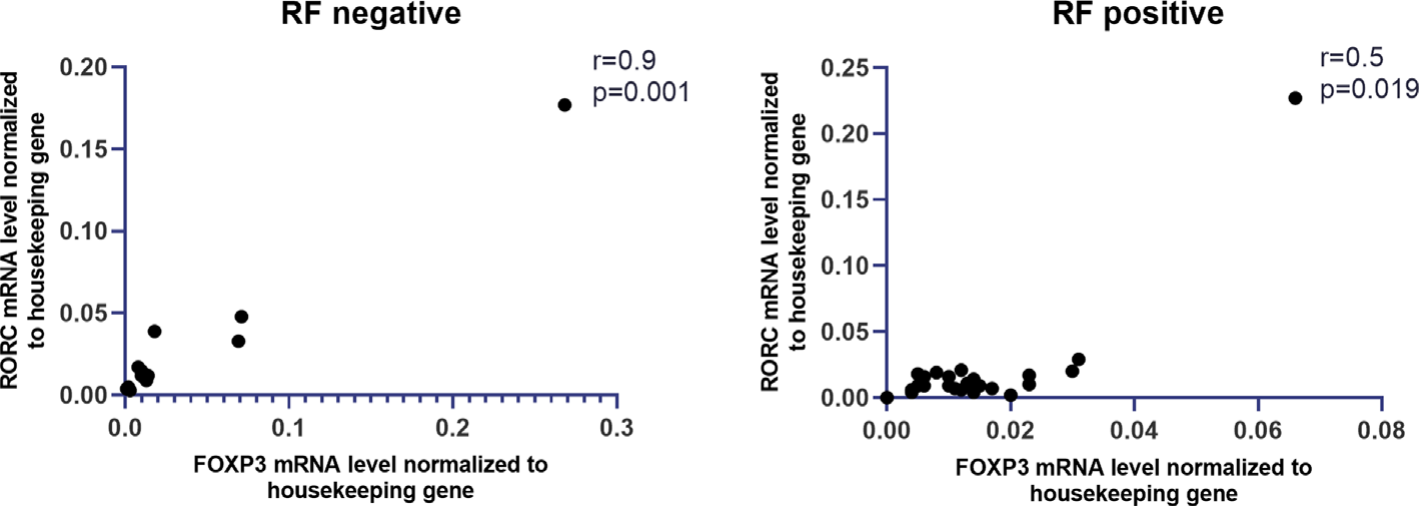
Correlation between RF presence and mRNA levels of FOXP3 and RORc in whole blood of RA patients.

The expression of all examined genes in whole blood and anti-CCP presence in RA also was investigated, but no significant correlation was found (**Figure 5S A in Supplementary Files**). We also observed that the expression of *STAT5a* in whole blood was higher in RA patients with rheumatoid factor (RF) compared with RA patients without RF, but the difference was not significant. *SOCS1* mRNA expression in whole blood was comparable between these two groups of RA patients (RF positive and RF negative). Furthermore, the other genes (*FOXP3, RORc, SMAD2-4, STAT3*) did not reveal statistically significant differences in RA patients with RF comparing to RA patients without RF (**Figure 5S B in Supplementary Files**). Interestingly, a significantly high correlation between expression of *FOXP3* and *RORc* was detected in RA patients without RF (r = 0.9, p = 0.001, **Figure 4**). This correlation was not detected in RA patients with RF. In contrast, in RA patients with RF, we showed a high correlation between expression of *STAT5a* and *SMAD2* and *SMAD4* (both r = 0.65, p = 0.0003, **Figure 6S in Supplementary Files**), whereas in RA patients without RF these correlations were not observed.

In Th17 and Treg cells, we did not note statistically significant differences in genes expression when compared RA patients with anti-CCP or RF and RA patients without anti-CCP or RF (**Figure 5S C–F in Supplementary Files**).

**Figure 5:**
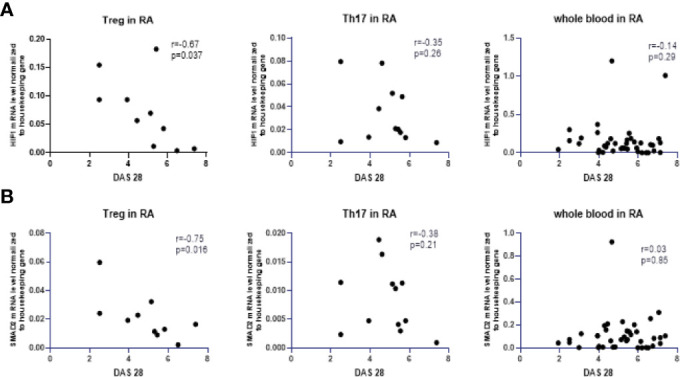
Correlation between DAS28 score and mRNA levels of HIF-1A **(A)** and SMAD2 **(B)** in RA Treg cells, in RA Th17 cells and whole blood of RA patients correlation with DAS28 score.

**Figure 6:**
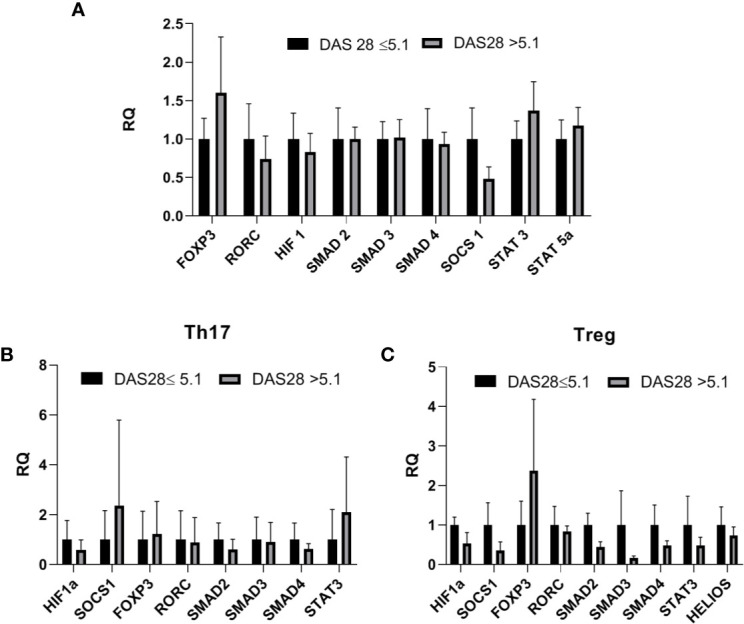
**(A)** Transcriptional factors mRNA level in whole blood in RA patients with DAS-28 >5.1 vs RA with DAS-28 ≤5.1 (relative expression in DAS-28 ≤5.1 was taken as 1), results are shown as mean ± SEM **(B, C)**. Th17/Treg-related transcriptional factors mRNA level in Th17/Treg cells from RA patients with DAS-28 >5.1 vs RA with DAS-28 ≤5.1. Data presented as mean ± SEM (relative expression in DAS-28 ≤5.1 was taken as 1).

Next, we analyzed the correlation between the expression of the examined transcriptional factors in whole blood and the DAS28 score in RA patients, however, these results are not significant (**Table 1S in Supplementary Files**). The correlation between the expression of examined transcriptional factors in RA Th17/Treg cells and the DAS-28 score was also examined (**Table 1S in Supplementary Files**). We found significant, negative correlations between expression of *HIF-1A* and *SMAD2* in RA Treg cells and DAS-28 score (r = −0.67, p = 0.037 and r = −0.7, p = 0.016, respectively, **Figure 5**). The expression of other transcriptional factors in Th17 or Treg cells were not associated with DAS-28 score. In RA patients with DAS-28 >5.1, *SOCS1*, and *STAT3* mRNA levels were lower in Treg cells and higher in Th17 cells, however, these differences were not significant (**Figure 6**). Excluding *FOXP3*, we observed a tendency for a lower expression of all analyzed genes in Treg cells from RA patients with DAS-28 >5.1. The expression of *FOXP3* was 3-times higher in RA patients with DAS-28 >5.1 when compared to RA patients with DAS-28 ≤5.1.

### Increased Serum Th17-Related Cytokines Levels in Patients With Rheumatoid Arthritis

**Figure 7:**
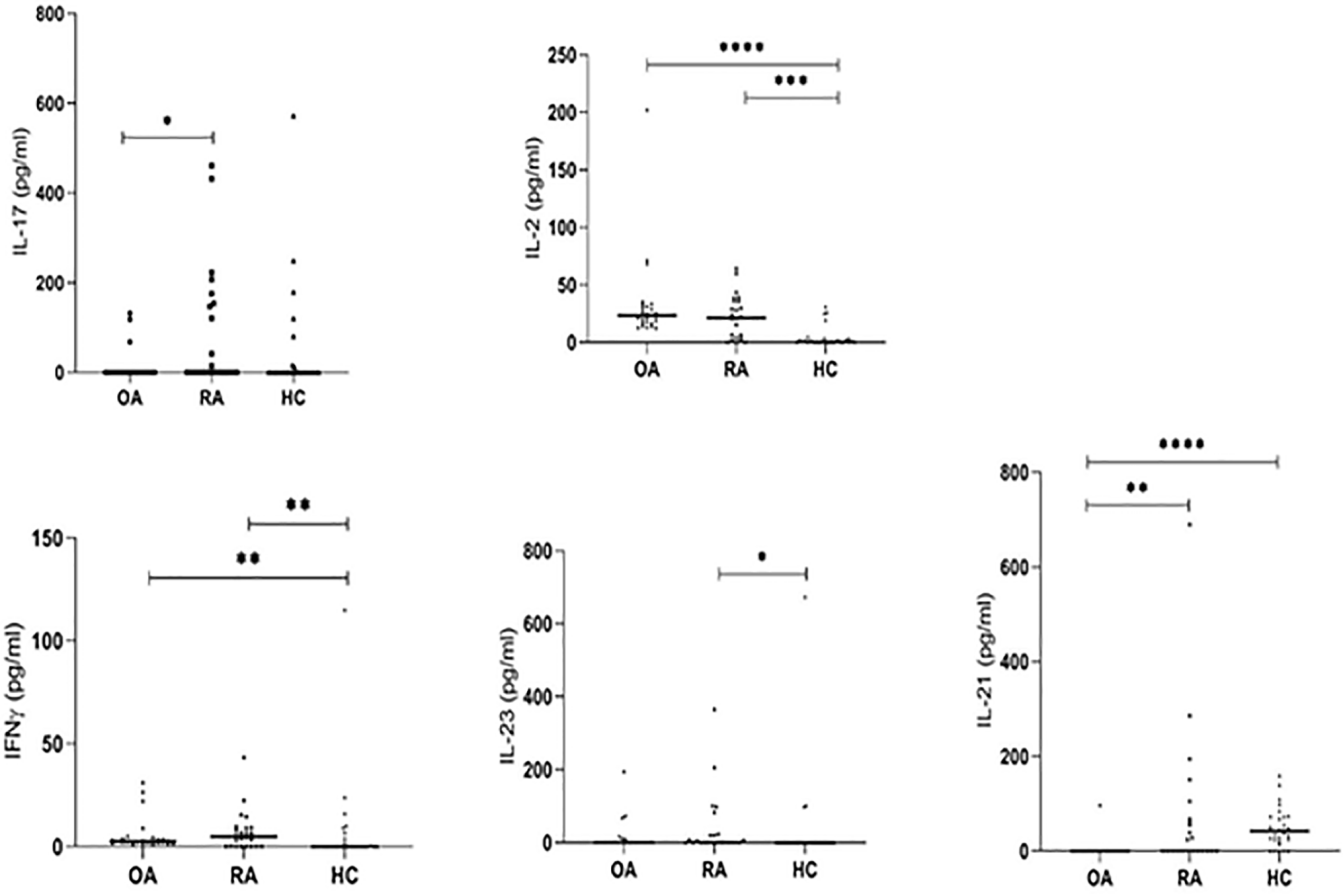
The serum IL-17, IL-21, IL-2, IL-23, IFN-γ levels detected by ELISA. *p < 0.05, **p < 0.01, ***p < 0.001, ****p < 0.0001.

To characterize the inflammatory environment contribution to the RA pathogenesis, we estimated the concentrations of different, Th17 or Treg-related cytokines (IL-17, IL-21, IL-22, IL-6, IL-10, IL-35, IL-2, IL-23, TGF-β, IFN-γ) in serum samples. The range of serum level of IL-17 and IL-21 was higher in RA patients than in OA patients (p = 0.024 and p = 0.006, respectively; **Figure 7**). The serum concentration of IL-2 and IFN-γ were significantly higher in RA and OA patients than in healthy subjects (in the case of IL-2 p = 0.0001 and p < 0.0001, respectively and both p = 0.007 in the case of IFN-γ; **Figure 7**). We also observed that serum levels of IL-21 were significantly higher in healthy subjects than in OA and RA patients (p < 0.0001 and p = 0.006, respectively; **Figure 7**). Serum concentration of IL-22, IL-6, IL-10, IL-35, and TGF-β were similar in all analyzed groups including healthy controls (**Figure 7S in Supplementary Files**).

In RA patients we revealed a significant correlation between analyzed serum cytokines levels. A positive correlation was demonstrated between IL-2 and IFN-γ (r = 0.9, p < 0.0001), IL-23 and IFN-γ (r = 0.92, p < 0.0001), IL-10 and IL-17F (r = 0.93, p < 0.0001), IL-17A and IL-17F (r = 0.8, p < 0.0001) as well as between IL-21 and IL-17F (r = 0.69, p < 0.0001) (**Figure 8S in Supplementary Files**). There were also weak but significant positive correlations between TGF-β and IL-2 (r = 0.59, p = 0.003), IL-22 and IL-17A (r = 0.59, p = 0.004), TGF-β and IFN-γ (r = 0.58, p = 0.003), and TGF-β and IL-23 (r = 0.47, p = 0.021).

In the present study, we also analyzed the correlation between transcriptional factors, mRNA expression and cytokine levels in serum. We observed a weak, but significant positive correlation between *STAT3* expression and serum IL-6 levels in RA patients (r = 0.4, p = 0.05, **Figure 9S in Supplementary Files**), and between *SMAD2* expression and serum IL-35 levels in OA patients (r = 0.47, p = 0.05, **Figure 9S in Supplementary Files**). Meanwhile, we did not find a significant correlation between the expression of other transcriptional factors and serum cytokines levels in both groups of patients as well as in healthy subjects.

### Correlation of Th17/Treg Ratio With Transcriptional Factors Expression, Cytokine Profiles, and Rheumatoid Arthritis Disease Activity

The relation between Th17 cells to Treg cells was estimated in RA patients, OA patients and healthy subjects by calculating the Th17/Treg ratio. In healthy subjects, a significant positive correlation between *SOCS1* expression and the Th17/Treg ratio was noted (r = 0.8, p = 0.002) (**Figure 10S in Supplementary Files**). In the case of OA patients, no significant correlation was found between the expression of the examined genes and the Th17/Treg ratio. On the other hand, in OA patients we found a weak, but significant positive correlation between serum IL-10 levels and Th17/Treg ratio (r = 59, p = 0.02). No significant correlation was found with OA patients clinical parameters such as patient age or mean value of CRP/ESR.

In RA patients, we did not find the correlation between the Th17/Treg ratio and the examined genes expression or the levels of cytokines in serum. We only observed that *STAT5a* expression increased with the increasing Th17/Treg ratio, however, this results were not significant. In the present study, we found no correlation between the Th17/Treg ratio and RA patients clinical parameters such as DAS-28 score, CRP/ESR, VAS score, Larsen score or disease duration.

### SMAD3 and STAT3 as Possible Diagnostic Biomarkers for Rheumatoid Arthritis

**Figure 8:**
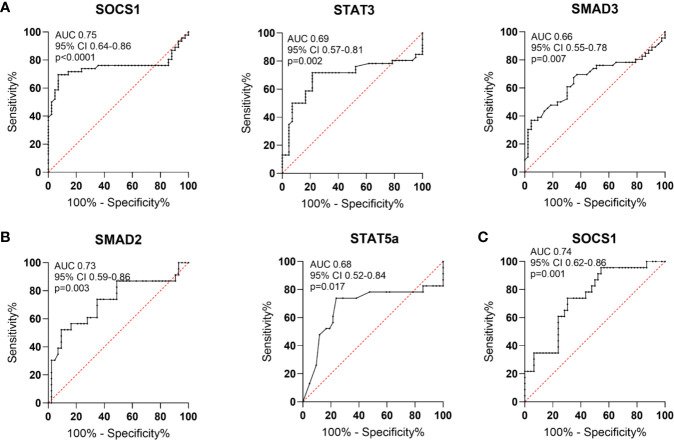
Schematic illustration of ROC curve to evaluate the diagnostic potential of AUC in whole blood **(A)**. in RA patients and healthy subjects **(B)**, in OA patients and healthy subjects **(C)**, in RA patients and OA patients.

Based on the receiver operating characteristics (ROC) curve analysis, the diagnostic usefulness of the relative level of mRNA of the analyzed genes in whole blood in the diagnosis of RA, OA as well as “differentiating” RA from OA was determined. Comparison between RA patients and healthy subjects has shown that the area under curve (AUC **Figure 8A**) was significant for *SOCS1* (AUC 0.75, p<0.0001), *STAT3* (AUC 0.69, p = 0.002), *SMAD3* (AUC 0.66, p = 0.007). Additionally, *STAT5a* (AUC 0.66, p = 0.017), *HIF-1A* (AUC 0.66, p = 0.01) and *RORc* (AUC 0.63, p = 0.03) revealed significant AUC (**Table 2S in Supplementary Files**). Comparison between OA patients and healthy subjects has shown that the AUC was significant for *FOXP3* (AUC 0.81, p<0.0001), *RORc* (AUC 0.73, p = 0.002) (**Table 3S in Supplementary Files**), *SMAD2* (AUC 0.73, p = 0.003) and *STAT5* (AUC 0.68, p = 0.02) (**Figure 8B**, **Table 3S in Supplementary Files**). Furthermore, the comparison between RA patients and OA patients has shown that the AUC was significant for *FOXP3* (AUC 0.75, p<0.0001), *SOCS1* (AUC 0.74, p = 0.001; **Figure 8C**), *SMAD3* (AUC 0.66, p = 0.03) and *RORc* (AUC 0.65, p = 0.05) (**Table 4S in Supplementary Files**).

**Figure 9:**
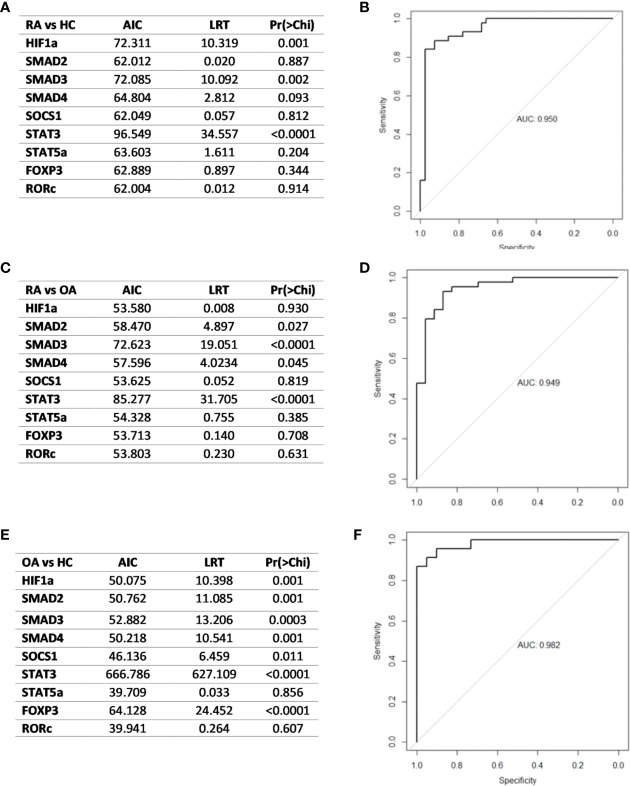
**(A)** Likelihood ratio test result in RA and healthy subjects **(B)** ROC curve and AUC value schematic representation of the logistic regression models for the combination of HIF1A, SMAD3 and STAT3-key in RA patients and healthy subjects differentiation **(C)**. Likelihood ratio test result in RA patients and OA **patients (D).** Logistic regression models for the combination of in RA patients and OA patients differentiation **(E)**. Likelihood ratio test result in OA and healthy subjects **(F)** logistic regression models for the combination of HIF-1A, SMAD2, SMAD3, SMAD4, SOCS1, STAT3 and FOXP3 in OA patients and healthy subjects. AIC, Akaike’s Information Criteria; LRT, Likelihood ratio test; Pr(>Chi), p-value corresponding to the Chi-square test compared to α = 0.05 level as significant.

In the next step, analyzed transcriptional factors were tested in a logistic model to found their diagnostic potential. Based on the Likelihood Ratio Test, we selected genes that were key in the logistic regression model in distinguishing RA patients from healthy subjects, RA patients from OA patients and OA patients from healthy subjects. Then the diagnostic value of the biomarker constituting the combination of the selected genes was estimated using ROC analysis. Based on the ROC analysis, the diagnostic potential of the combination of the genes, *HIF1*α, *SMAD3* and *STAT3*, was determined at AUC 0.95 for distinguishing RA patients from healthy subjects (**Figures 9A, B**). For distinguishing RA patients from OA patients the diagnostic potential of the combination of *SMAD2*, *SMAD3*, *SMAD4* and *STAT3*, was determined at AUC 0.95 (**Figures 9C, D**). For distinguishing OA patients from healthy subjects the diagnostic potential of the combination of the *HIF1*α, *SMAD2*, *SMAD3*, *SMAD4*, *SOCS1*, *STAT3*, and *FOXP3*, was determined at AUC 0.98 (**Figures 9E, F**).

## Discussion

Numerous transcriptional factors may regulate the interplay between immune suppression (Treg) and autoimmunity (Th17) in RA patients. The current state of our knowledge in this area is far from being complete and further investigations are needed to reveal a better understanding of the transcriptional factors expression role in the regulation of the Th17/Treg balance and RA susceptibility and severity. Our earlier studies (15) have shown that in the same group of RA patients, as presented in the current manuscript, some relationships exist between miRNA and transcriptional factors mRNA expression in the Th17 and Treg cells. We observed that the expression of miR-26 was positively correlated with *SMAD3*, *STAT3* and *SOCS1* expression as well as expression of miR-155 was positively correlated with *STAT3* expression in RA Th17 cells. In RA Treg cells a positive correlations were observed between miR-26 and *SOCS1*, miR-31 and *SMAD3* and miR-155 and *SMAD3/SMAD4*. In contrast, in RA Treg cells a negative correlation was observed between miR-26 and *STAT5A* (15).

To our knowledge, the current study, which is a continuation of our earlier research (15), for the first time has compared the Th17/Treg-related transcriptional factors mRNA expression profile in the whole blood with the expression profile in Th17 and Treg cells. The main findings of the present study were that 1) *SMAD3* and *STAT3* may have the highest diagnostic potential in RA patients classification, 2) positive correlation between *SMAD3* and *STAT3* in RA patients was observed, 3) *STAT5a* expression was not detected in Th17 cells, 4) Treg/Th17 ratio index in RA patients’ blood was lower than in HC blood, clearly indicating a disturbance in Th response, 5) the range of IL-17 and IL-21 serum levels were higher in RA patients than in OA patients as well as higher serum IL-2 and IFN-γ levels in RA and OA patients than in healthy subjects.

In the present study, we have confirmed results of some previous studies showing INCREASED Th17 cells frequency, as well as Th17/Treg ratio in RA patients (16–22). In contrast to our study, Penatti A et al. (23) have shown that RA patients had lower frequencies of circulating CCR6+CXCR3−Th17 cells and higher frequencies of conventional CD25+ Tregs comparing to the OA patients. These differences could be due to the higher disease activity and corresponding higher doses of the disease-modifying anti-rheumatic drugs (DMARDs) (methotrexate (15–25 mg/week) or leflunomide (20 mg/day) and glucocorticoids (GCs) in our RA patients. Tada Y et al. (24) postulated that *FOXP3* expression may be also modulated by the used therapy. They found that *FOXP3* expression and the *FOXP3/ROR-γt* expression ratios were increased after tocilizumab therapy. Furthermore, Cribbs AP et al. (25) found that MTX therapy acts specifically at the *FOXP3* upstream enhancer region to increase *FOXP3* expression. Both, Tada Y et al., and Cribbs AP et al. did not compare DMARDs therapy with biological therapy. Earlier studies (26) have also shown that GCs treatment diminished IL-17 levels, which is in agreement with our observations. We observed that the serum level of IL-17 was higher in RA patients than in healthy subjects, nevertheless, the difference was not significant. But on the other hand, a pathogenic inflammatory Th17 phenotype may cause resistance to GCs leading to high disease activity in our RA patients. In line with previous studies, we also observed that the range of serum of IL-17 and IL-21 levels were higher in patients with RA compared to OA patients as well as serum IL-2 and IFN-γ levels were higher in RA patients than in healthy subjects (3, 12, 27–30)). The role and biological functions of IL-17 and IL-23 in the RA pathogenesis were very carefully described in our earlier papers (31, 32). Interestingly, IL-21 and IL-23 activate *STAT3*, which is essential for the differentiation of the Th17 cells (33). Whereas, the production of IL-23 is inhibited by IFN-γ, at the same time it is stimulated by IL-6, *STAT3* and *RORc* (34). Therefore, our data suggest that the reduced expression of some transcriptional factors, as well as lower serum cytokine levels observed in RA patients, might be the result of using therapy, which is a crucial factor that should be taken into account in further studies. We and other authors have confirmed that *SOCS1* is highly expressed in Treg cells (35) as well as that *SOCS1* may be involved in the Th17 cell differentiation and function (36). On the one hand, overexpression of *SOCS1* in whole blood observed in the present study may reflect the ongoing inflammatory process. On the other, it may show that the immune system is trying to overcome the ongoing inflammatory process (37, 38), as upregulated *SOCS1* expression may have inhibitory effects on arthritis development. In RA patients with active disease, increased *SOCS1* mRNA expression can inhibit the cytokine signaling pathways and potentially prevents the harmful effects of proinflammatory cytokines. However, high serum SOCS1 levels can also suppress the beneficial actions of anti-inflammatory cytokines leading to the breakage of immunoregulatory mechanisms in RA.

In the present report, we also demonstrated that RA Treg cells revealed very high *HELIOS* and *FOXP3* expression levels, which indicates that there is an enrichment for Tregs. Interestingly, P. Zafari et al. (39) suggested that increased *FOXP3* gene methylation and increased *HELIOS* gene expression in whole blood may play an important role in the RA pathogenesis through their effects on promoting Treg’s stability. Moreover, Yang et al. (14) postulated that *HELIOS* may play an essential role in Treg immunosuppressive function in RA patients, especially in RA patients with high disease activity, as it suppresses Treg’s ability to express effector cytokines. Although we observed higher Treg cells frequency in OA patients than in RA patients, *HELIOS* gene expression was higher in RA patients than in OA. The reason might be explained by different OA and RA etiopathology. While OA is a predominantly degenerative disease, RA is an autoimmune disease mainly driven by a significant inflammatory response involving both innate and adaptive immune systems (23). The presence of Treg cells in both RA and OA patients may be explained as an attempt by the immune system to control the inflammatory responses (23). However, the much more active disease in RA patients could be due to the complicated inflammatory environment, which might drive to inappropriate Treg cells function.

In our study, except in the case of *STAT5A* and *SMAD2*, we did not observe differences in the expression of the analyzed genes between RA patients treated with MTX and RA patients treated with biological therapy. mRNA levels of *STAT5A* and *SMAD2 *were higher in RA patients treated with the biological therapy (**Figure 11S in Supplementary Files**). To the best of our knowledge, the present study is the first showing analysis of *SMAD2* expression in RA patients in relation to used therapy. Moreover, we also observed downregulated *SMAD2* mRNA levels in RA/OA Th17 cells in comparison to Treg cells. It is an interesting finding in relation to the earlier studies showing that SMAD2 acts as a positive regulator of Th17 differentiation (33, 40). In Th17 cells, phosphorylated *SMAD2* acts as a *STAT3* co-activator leading to the Th17 cell differentiation, and probably that *SMAD2* induces an active chromatin state for Th17 regulation. Our results indicated that Tregs may be possibly skewed toward IL-17–producing Treg cells or this may be an effect of immunosuppressive therapy which is used in most of our RA patients. However, further studies are required to elucidate the detailed molecular role of SMADs in Th17 differentiation. In addition, in Treg cells, *STAT5*, a key positive regulator of *FOXP3* (6, 41), revealed a very low expression level in all examined groups. In contrast, in the whole blood, we observed a very high *STAT5* mRNA level in RA patients, but in OA patients *STAT5* expression level was comparable with this noted in the healthy subjects. It may be the result of neutrophils and eosinophils presence in the whole blood, which express both STAT5A and STAT5B isoforms (42).

Considering that all examined genes are important transcriptional factors in Th17/Treg balance, we also compared genes expression and their correlation with RA activity. However, we do not observe a significant correlation between examined transcriptional factors and RA activity. We only noticed a tendency that *SOCS1* and *STAT3* expressions were lower in Treg cells and at the same time higher in Th17 cells in RA patients with DAS-28 >5.1, but the differences were not significant. The lack of correlations between examined transcriptional factors expression and RA activity may be explained by relatively long disease duration and medication, which may overshadow existing interactions. Both MTX and biological therapy may have an impact on the gene expression as well as the proportion and absolute numbers of Th17 and Treg cells. Interestingly, we also observed a negative, although not significant, the correlation between the expression of *HIF-1A* in RA Treg cells and DAS-28 score. Although HIF-1A was originally discovered as a hypoxia sensor, today it is estimated that *HIF-1A* gene expression, as well as HIF-1A protein levels, maybe also affected by other relevant factors including reactive oxygen species, leading to HIF-1A proteasomal degradation (43).

One of the objectives of the study was to find a biomarker to distinguish RA and OA. The detection of novel blood biomarkers that could serve for the classification and/or monitoring of the disease progression would be of great importance for RA patients. Peripheral blood is an available and informative source of markers for several diseases. The analysis of gene expression in whole blood may be an informative method used to investigate disease states, examine immune responses as well as identifying biomarkers. The data selected based on the results, which were obtained from the likelihood ratio test, revealed that *SMAD3* and *STAT3* may be possible diagnostic biomarkers for rheumatoid arthritis. Alikhah A et al. (44) evaluated the expression level of *STAT3* as the most important transcription factor in Th17 cell development because it is engaged in the control of the inflammatory processes. High *STAT3* expression level may stimulate *ROR*c as well as inhibit *STAT5-FOXP3* binding which leads to the destabilization of Treg cells and promotes Th17 differentiation and their function (45). It was reported that STAT3 through the regulation of the proinflammatory pathway, which is important in the RA pathogenesis, is required for the receptor activator of NF-κB (RANK) ligand induction, osteoclast formation, chemokines production and synovitis (46, 47). In addition, previous reports indicated that *STAT3* may regulate the *HIF1α* gene transcription and promotes HIF1α protein stability (47, 48). Single nucleotide polymorphisms (SNPs) located in the *STAT3*-binding motif could result in either gain or loss of *STAT3* binding. SNPs at position rs3024505, rs947474, rs6580224, and rs2293607 (mutant allele) alter *STAT3* binding to target genes and suggest that polymorphisms may regulate *STAT3*-mediated transcription by affecting the expression of factors that are significant for early Th17 cell differentiation. Besides, patients with mutations classified as “gain-of-function” in *STAT3* gene had an increased Th17 frequency and a diminished number of Tregs, and this dysregulation of the immune system could explain their autoimmune diseases (49, 50). Moreover, hyperactivation of *STAT3* and at the same time lack of *STAT5* expression in Treg cells, what was also observed in our study, may simplify the conversion of Treg cells to Th17-like cells, causing an uncontrolled inflammatory response (51, 52). Furthermore, *SMAD3*, which plays a critical role in the joint homeostasis and repair of cartilage, is one of the key downstream mediators of the transforming growth factor-β (TGF-β) signaling pathway. However, *SMAD3* gene expression may be also affected by mutations/SNPs, which lead to the dysregulation of the TGF-β pathway. Arg287Trp mutation creates a protein that is unable to form SMAD3 protein homomers or heteromers with SMAD4, attenuate TGF-β signaling and this may lead to an even greater reduction in functional SMAD3, and therefore a more severe disease phenotype (53). The crosstalk between *SMAD3* and *STAT3* may regulate SMAD3 functions, suggesting that one or more unknown factors determine the outcomes of their cooperation. Some studies suggested that the peripheral T regulatory 17 (Tr17) cells are more regulatory than classical Tregs (54, 55). Tr17 cells are the activated Treg cells that regulate Th17 cell-dependent autoimmunity in a STAT3-dependent manner. Inhibition of STAT3 activity may probably be sufficient to block inflammation and osteoclast activities in joints. Longitudinal studies would be valuable to determine a diagnostic value of these factors. A better understanding of the *SMAD3–STAT3* interaction would help us to find the molecular background of RA and improve therapeutic strategies against inflammatory diseases. Moreover, gene expression analyzed in whole blood may also be influenced by different blood parameters, which varies in individuals or in disease states.

Our study has some limitations. The first is that cells were not sorted immediately after isolation from blood, but after overnight culture. It should be taken into account that the dying cells during the overnight culture secrete various substances that may affect the activation status of other cells. In our experiments, the viability was high (around 95%), but the influence of this mechanism on the results obtained by us cannot be ruled out. Due to the gating strategy we have used, the small Th17 contamination in the Treg population cannot be excluded. Second, in case of age, we have noted significant differences between groups. First of all, blood donors are limited to their age, and second, our OA group was aged than 60 years old patients. Therefore, we adjusted gene expression in whole blood by age. Thirdly, most of our RA patients were on high doses of immunosuppressive therapy, which have a very strong immunomodulatory effect, reduces inflammation, and which may influence gene expression that lead to a lack of correlation between examined genes and RA activity.

In conclusion, our data indicated that *SMAD3* and *STAT3* may have the highest diagnostic potential in RA patients classification. Moreover, we believe that the presented gene expression profile is another stage in understanding the basic biology/pathogenesis of rheumatoid arthritis and it may lead to more rational treatment strategies. Therefore, the anti-rheumatic drugs and the background of the treatment can affect the results of our study. We suggest that the background of therapy may be a critical factor disturbing the Th17/Treg balance that should be taken into account when planning future clinical studies. Therefore, untreated RA patients should be compared with MTX and/or biologics treated RA patients in further/future study. Identification of specific biomarkers/factors involved in regulation of Th17/Treg balance is important not only in the case of RA but also other various inflammatory and autoimmune diseases. Additionally, the specific biomarkers may also be useful for the diagnosis, classification, prognosis of diseases and prediction of the therapeutic response. Moreover, the expected results will provide knowledge to be used in the future for clinical trials.

## Data Availability Statement

The data sets presented in this study can be found in online repositories. The names of the repository/repositories and accession number(s) can be found in the article/**Supplementary Material**.

## Ethics Statement

The studies involving human participants were reviewed and approved by Ethics Committee of the National Institute of Geriatrics, Rheumatology, and Rehabilitation, Warsaw, Poland (approval protocol number 29 June 2016). Written informed consent to participate in this study was provided by the participants’ legal guardian/next of kin.

## Author Contributions

AP-G conceived, designed, and performed the experiments, analyzed and interpreted the data, and wrote the first draft of the manuscript. AW performed the data analysis and contributed to drafting of the manuscript; EW, EK-W, AW, TK, BS, and ER performed experiments; PJ and AP contributed to drafting of the manuscript, KR-P and DM were involved in classification of patients with RA and OA, clinical check of patients and treatment control. All authors contributed to the article and approved the submitted version.

## Funding

This work was supported by grant from the Polish National Science Center 2015/B/NZ5/00247.

## Conflict of Interest

The authors declare that the research was conducted in the absence of any commercial or financial relationships that could be construed as a potential conflict of interest.

## Abbreviations

RA, rheumatoid arthritis; OA, osteoarthritis; HCs, healthy controls; RORc, retinoic acid receptor-related orphan receptor variant 2; FOXP3, forkhead transcription factor; STAT3, signal transducers and activators of transcription; SMAD2, Sma- and Mad-related protein2; SMAD3, Sma- and Mad-related protein 3; HELIOS, Ikaros family zinc-finger protein 2 (IKZF2); HIF-1A, hypoxia-inducible factor 1α; SOCS, suppressor of cytokine signaling; CRP, C-reactive protein; ESR, erythrocyte sedimentation ratio; RF, rheumatoid factors; anti-CCP, anti-cyclic citrullinated peptide autoantibodies; DAS-28, disease activity score in 28 joints; VAS, visual analog scale; qPCR, quantitative real-time PCR; ELISA, enzyme-linked immunosorbent assay; ROC, receiver operating characteristics; AUC, area under curve; COX-2, cyclooxygenase-2; PGE2, prostaglandin E2.
