# Supplementary material for: Th17/Treg-Related Transcriptional Factor Expression and Cytokine Profile in Patients With Rheumatoid Arthritis
Source: Front Immunol. 2020 Dec 11;11:572858. doi: 10.3389/fimmu.2020.572858 (PMC7759671; doi:10.3389/fimmu.2020.572858)
Supplement: Supplementary file 1 [file DataSheet_1.doc]

Supplementary Material


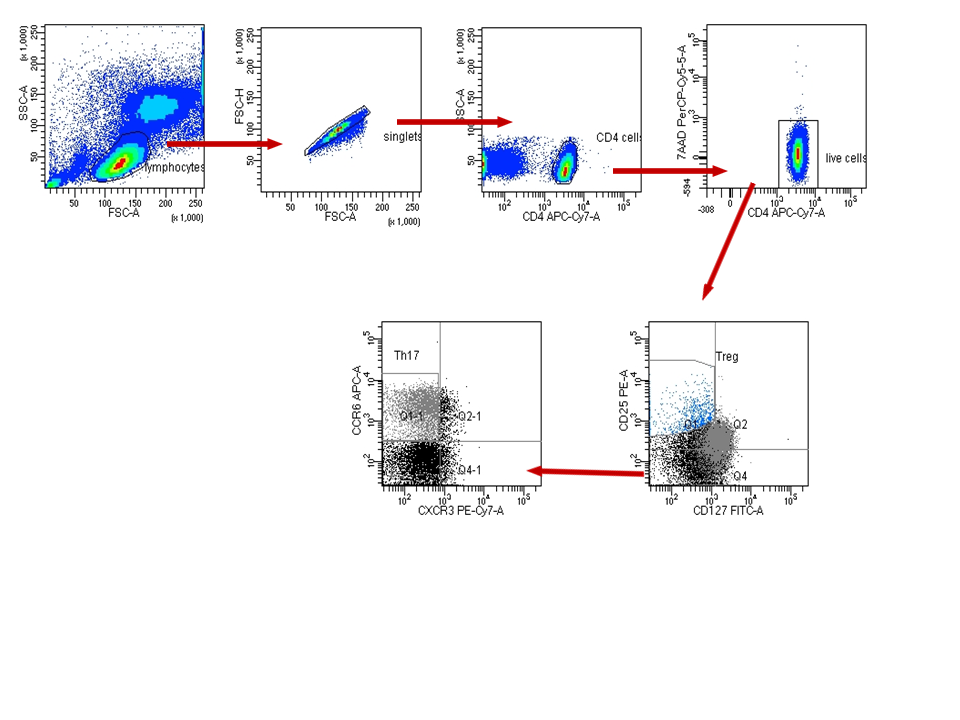


**Figure 1S .** Representative gating strategy for FACS of PBMC, showing Treg and Th17 lymphocyte.


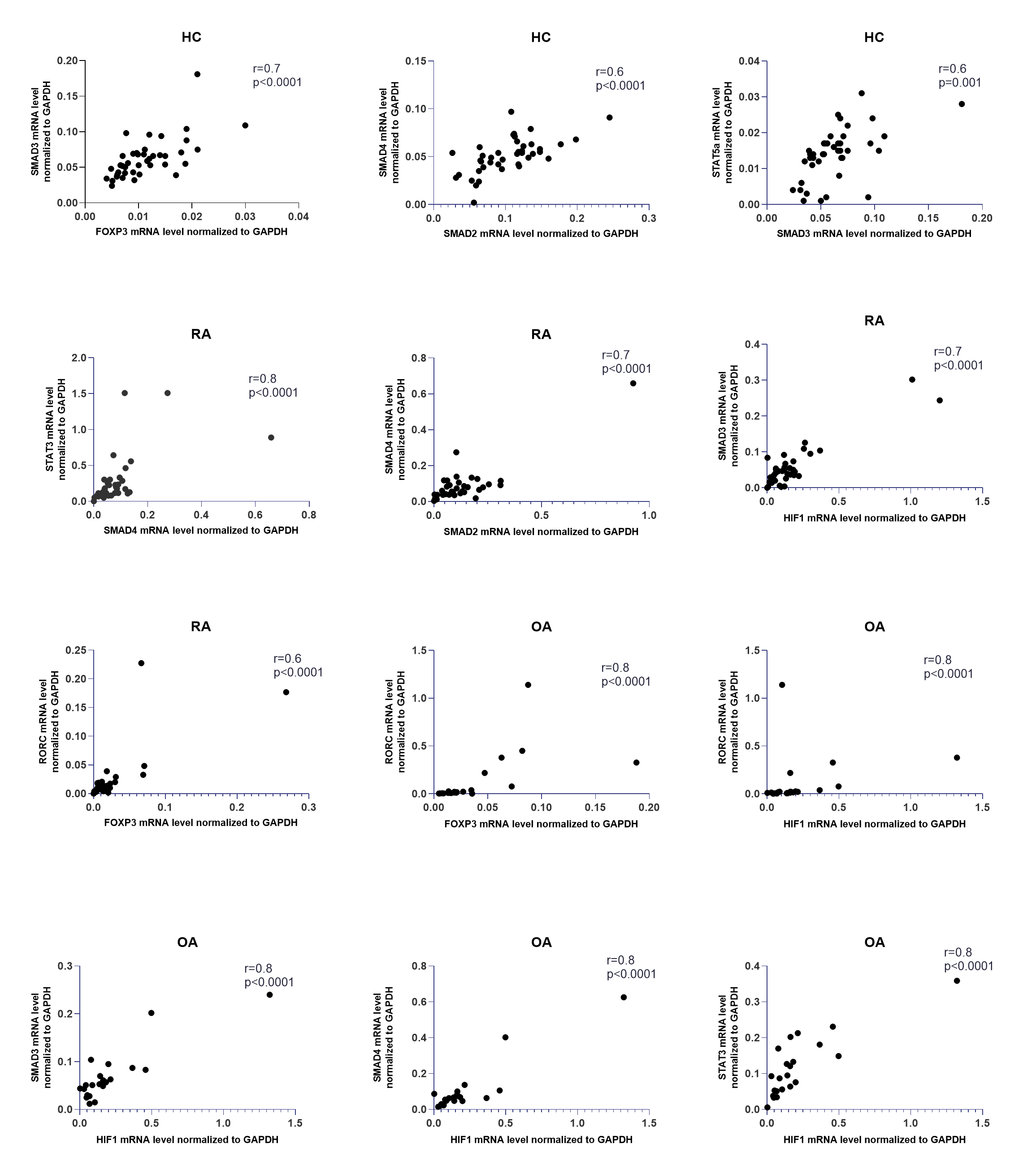


Figure 2S. The correlation between examined genes expression in blood in healthy subjects (HC), RA and OA.


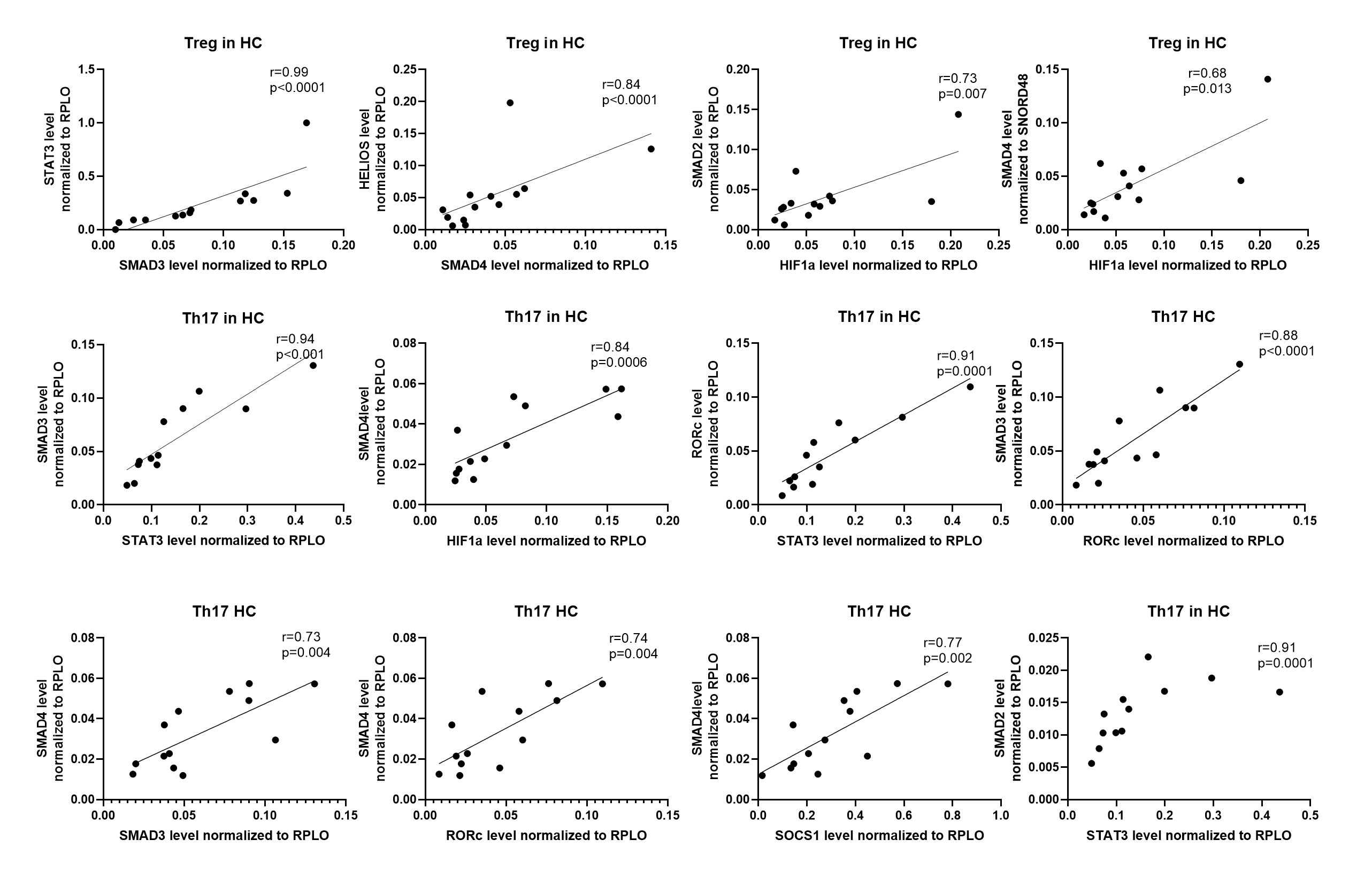


**Figure 3S.** The correlation between examined transcriptional factors in Treg cells and Th17 cells isolated from healthy subject.


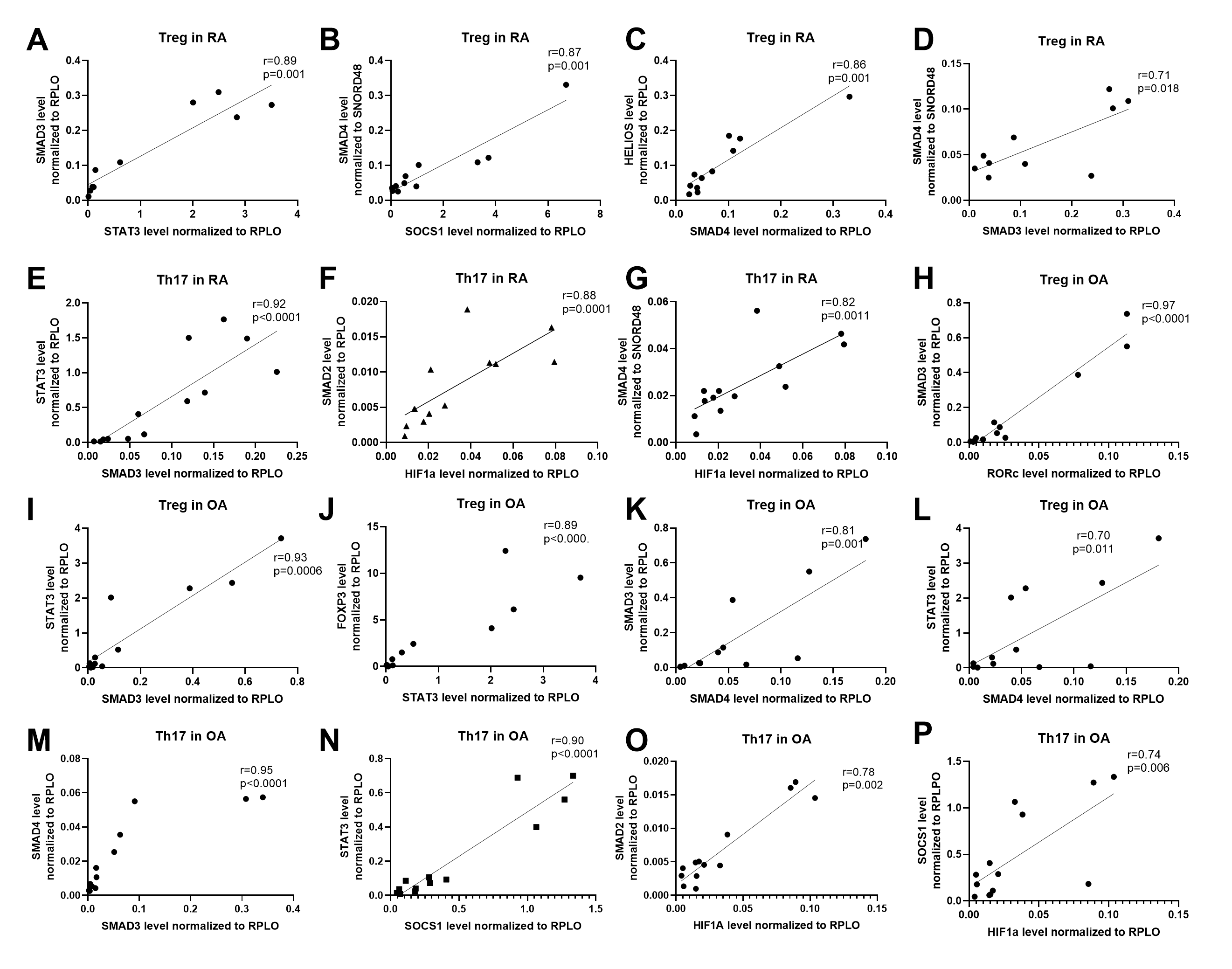


**Figure 4S.** The correlation between transcriptional factors in Treg cells (A-D) and Th17 cells (E-G) isolated from RA patients, Treg cells (H-L) and Th17 (M-P) isolated from OA patients.


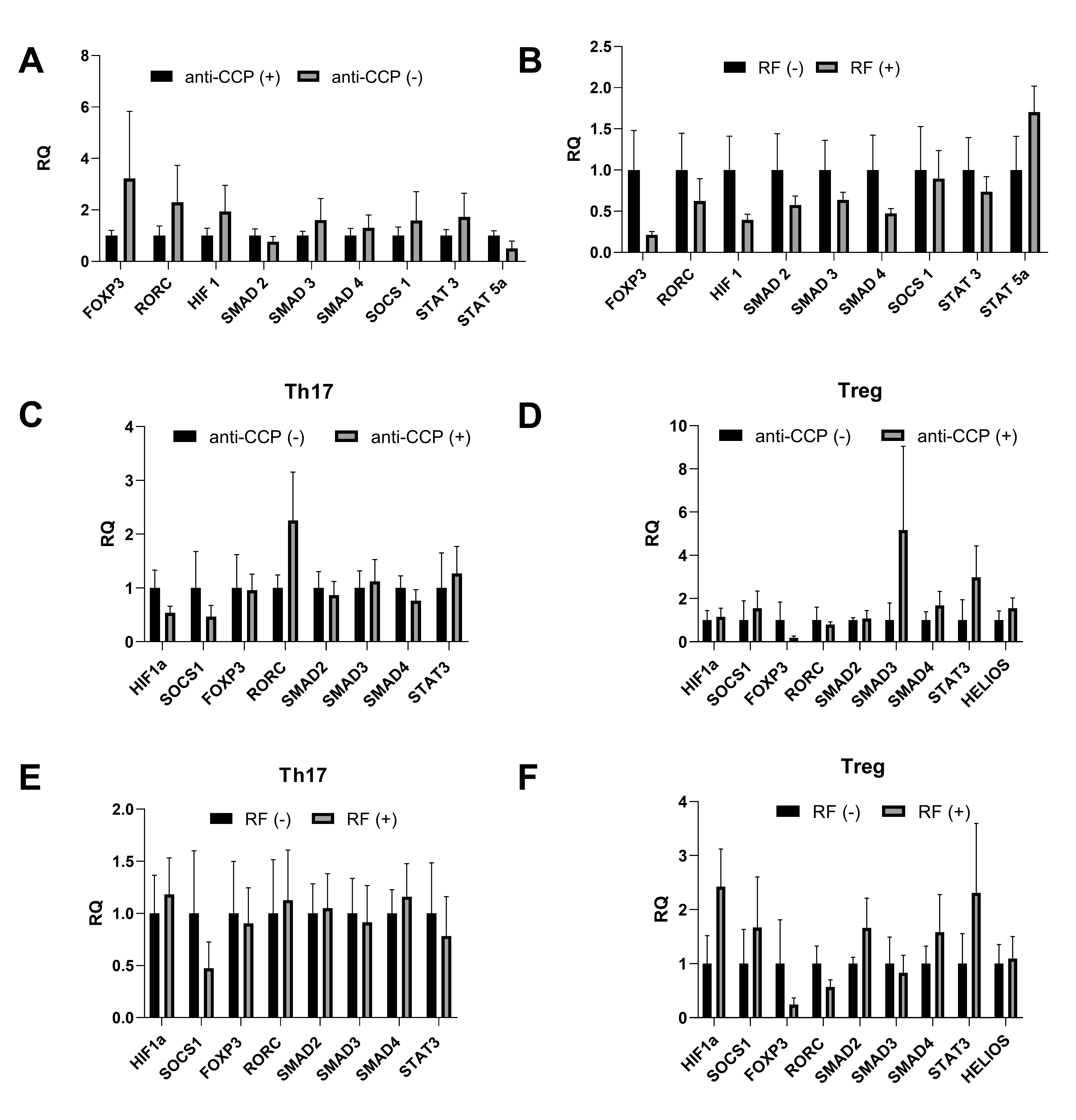


**Figure 5S.** **(A)** Transcriptional factors mRNA level in whole blood in RA patients with anti-CCP positive (aCCP+) vs RA with anti-CCP negative (aCCP-). (relative experession in anti-CCP(+) was taken as 1),results are shown as mean±SEM. **(B)**Transcriptional factors mRNA level in whole blood in RA patients with RF positive (RF+) vs RA patients with RF negative (RF-) (relative experssion in RF- was taken as 1), results are shown as mean±SEM. **(C-F)** Th17/Treg-related transcriptional factors mRNA level in Th17/Treg cells from RA patients with anti-CCP /without anti-CCP, from RA patients with RF/without RF. Data presented as mean±SEM. (relative expression in ani-CCP (-) and RF (-) was taken as 1).


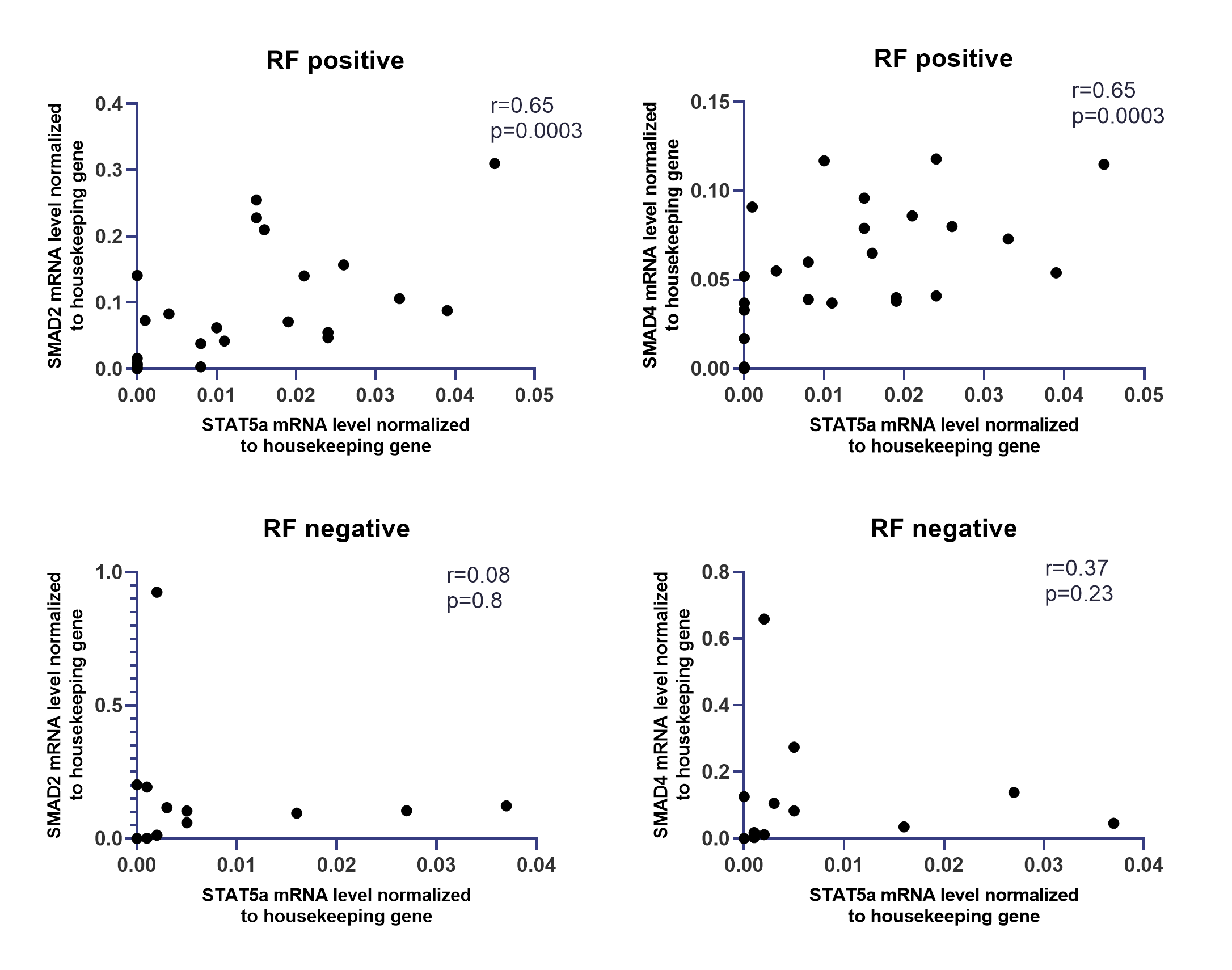


**Figure 6S.** Correlation between RF presence and mRNA levels of STAT5a and SMAD2 and SMAD4 in whole blood of RA patients.

**Table 1S.** The correlation between examined gene expression and DAS28 score in patients with RA

| **Whole Blood** | **DAS 28 vs. FOXp3** | **DAS 28 vs. RORC** | **DAS 28 vs. HIF 1** | **DAS 28 vs. SMAD 2** | **DAS 28 vs. SMAD 3** | **DAS 28 vs. SMAD 4** | **DAS 28 vs. SOCS 1** | **DAS 28 vs. STAT 3** | **DAS 28 vs. STAT 5a** |  |
| --- | --- | --- | --- | --- | --- | --- | --- | --- | --- | --- |
| **r** | 0,07659 | -0,0805 | -0,1748 | 0,03209 | -0,1356 | 0,02369 | -0,1746 | 0,004859 | -0,1257 |  |
| **p-value** | 0,6523 | 0,6358 | 0,2871 | 0,8462 | 0,4103 | 0,8862 | 0,2877 | 0,9766 | 0,4457 |  |
| **Treg cells** | **DAS 28 vs. HIF1a** | **DAS 28 vs. SOCS1** | **DAS 28 vs. FODAS 28P3** | **DAS 28 vs. RORC** | **DAS 28 vs. SMAD2** | **DAS 28 vs. SMAD3** | **DAS 28 vs. SMAD4** | **DAS 28 vs. STAT3** | **DAS 28 vs. STAT5a** | **DAS 28 vs. HELIOS** |
| **r** | **-0,6748** | -0,31 | 0,5471 | 0,1033 | **-0,7477** | 0,01674 | -0,2675 | 0,09726 | -0,3343 | 0,1033 |
| **p-value** | **0,0372** | 0,3796 | 0,1064 | 0,7769 | **0,0164** | 0,9733 | 0,452 | 0,7911 | 0,3416 | 0,7769 |
| **Th17 cells** | **DAS 28 vs. HIF1a** | **DAS 28 vs. SOCS1** | **DAS 28 vs. FODAS 28P3** | **DAS 28 vs. RORC** | **DAS 28 vs. SMAD2** | **DAS 28 vs. SMAD3** | **DAS 28 vs. SMAD4** | **DAS 28 vs. STAT3** | **DAS 28 vs. STAT5a** | **DAS 28 vs. HELIOS** |
| **r** | -0,3503 | 0,09107 | 0,1576 | 0,1086 | -0,3853 | 0,1471 | -0,1786 | 0,2242 | -0,4869 | -0,1681 |
| **p-value** | 0,2623 | 0,7781 | 0,6227 | 0,7366 | 0,215 | 0,6459 | 0,5761 | 0,4801 | 0,1107 | 0,5986 |

Red color means that correlation is high and statistically significant.


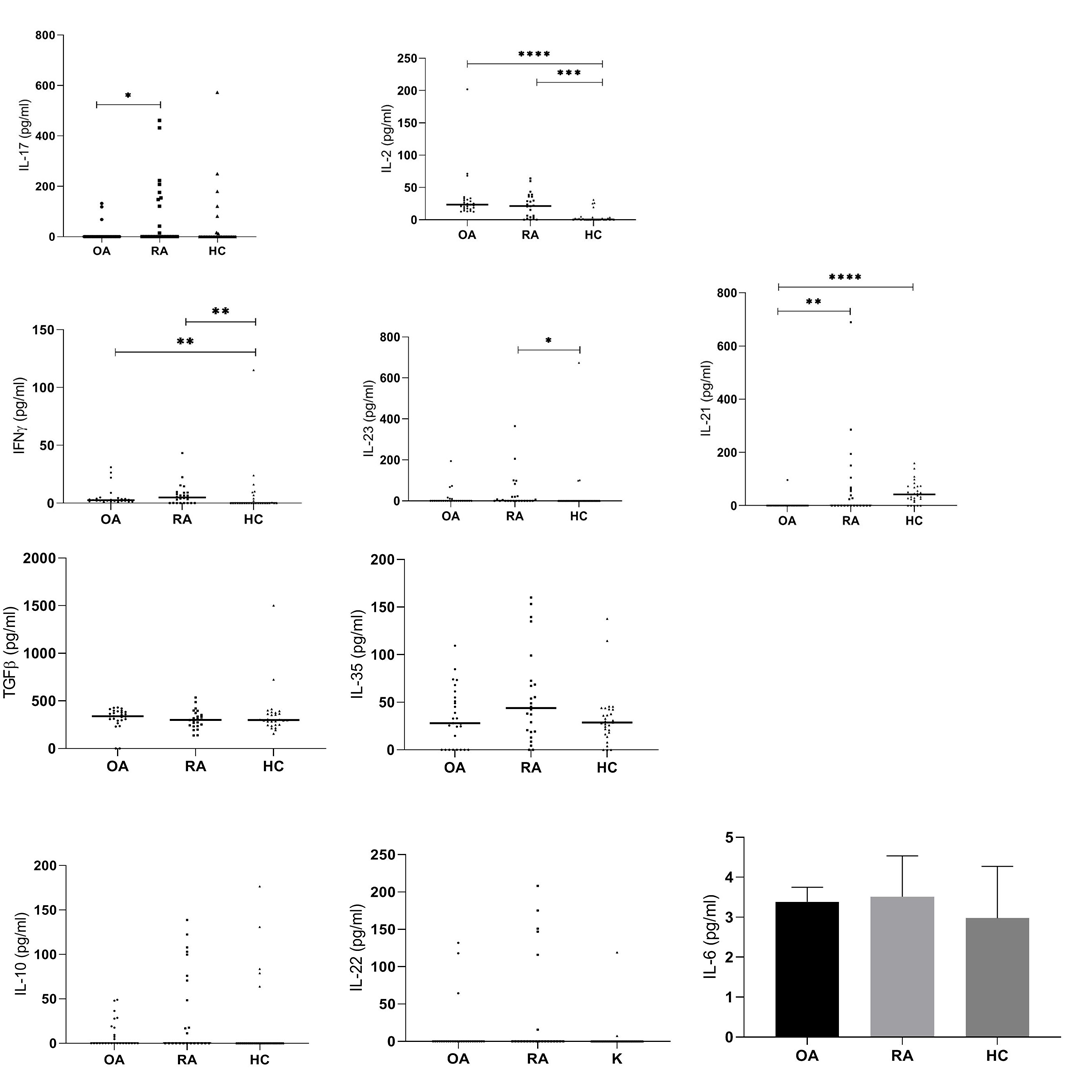


**Figure 7S.** The serum IL-22, IL-6, IL-10, IL-35, TGF-β, levels detected by ELISA.*p<0.01, **p<0.001, *** p<0.0001, ****p<0.00001.


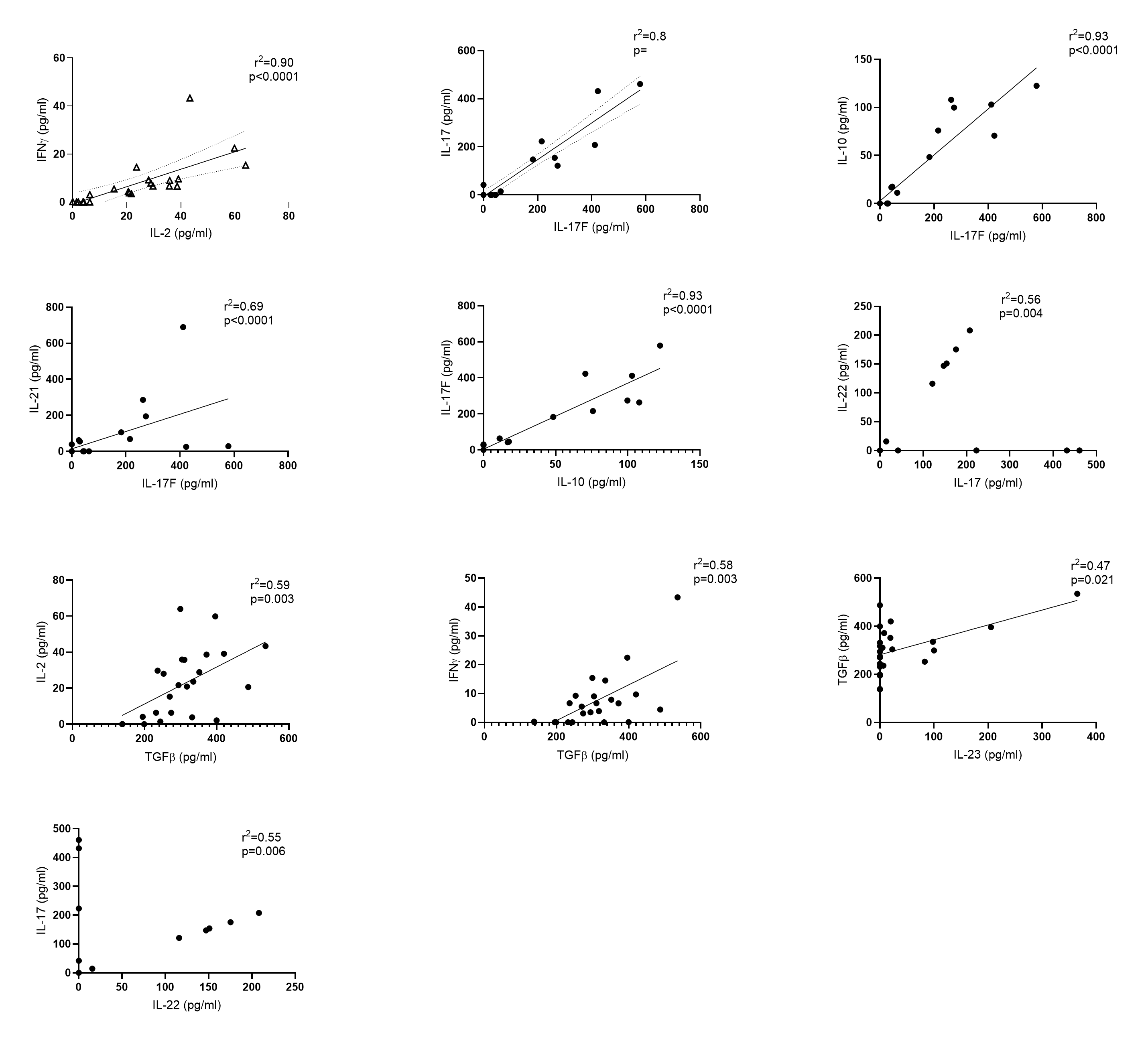


**Figure 8S.** A high, positive correlation between examined cytokine in serum of RA patients.

**Figure 9S.** Positive correlation between STAT3 expression and serum IL-6 levels in RA patients, and between SMAD2 expression and serum IL-35 levels in OA patients.


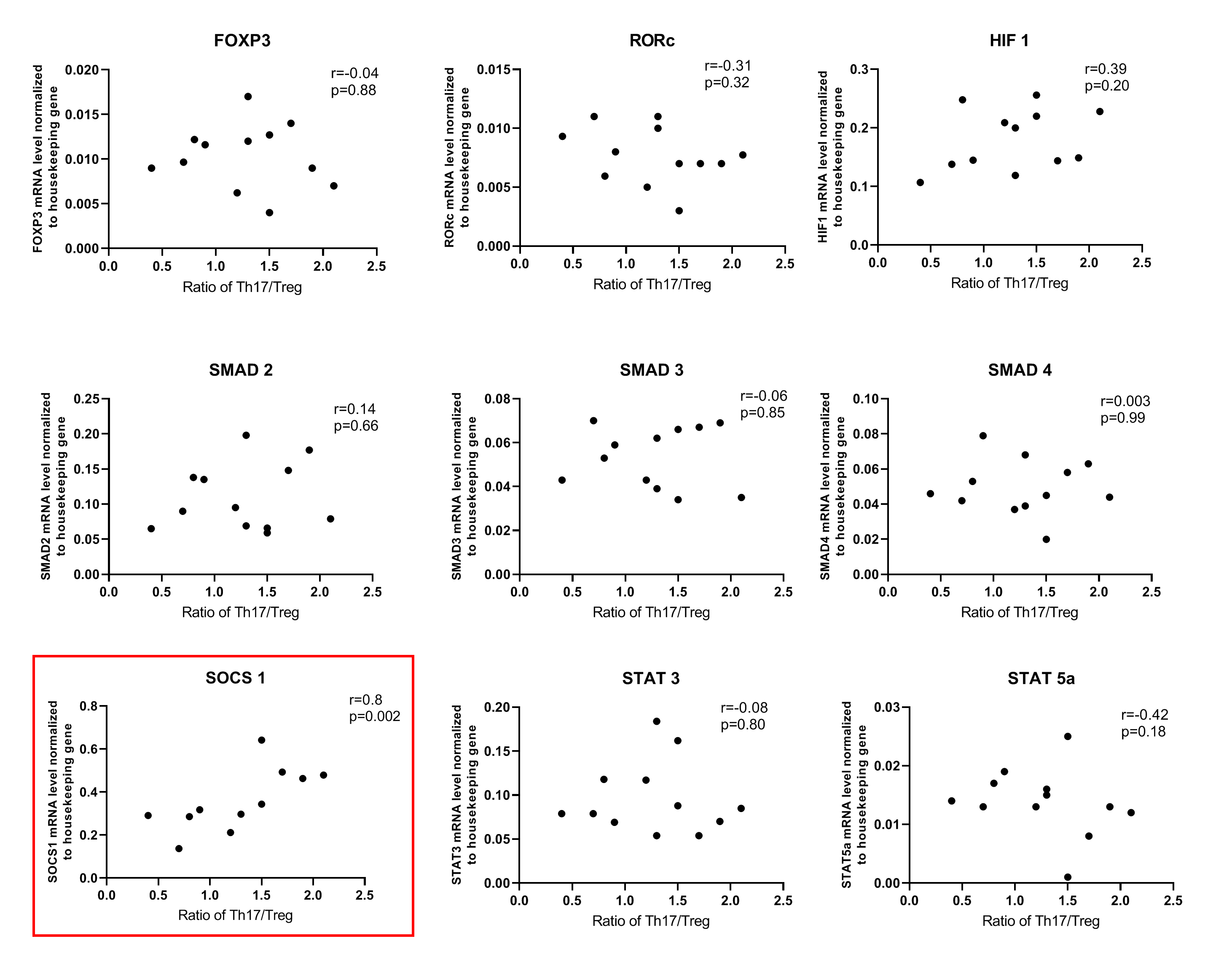


**Figure 10S.** Correlations of the relative level of genes expression and the Th17/Treg ratio in healthy subjects.

**Table 2S.** Relative expression level of examined genes in RA patients and healthy subjects (HC) based on the area under the ROC - AUC (Area Under Curve) curve.

| **GEN** | **mediana (IQR)** | **AUC** | **p*** |
| --- | --- | --- | --- |
| **SOCS1** |  |  |  |
| **HC** | 0.02(0.0-11.53) | 0.75 | <0.0001 |
| **RA** | 0.17(0.0-18.32) |  |  |
| **STAT3** |  |  |  |
| **HC** | 0.34(0.0-57,42) | 0.69 | 0.002 |
| **RA** | 2.78(0.0-63.54) |  |  |
| **SMAD3** |  |  |  |
| **HC** | 0.0014(0.0-3.304) | 0.66 | 0.007 |
| **RA** | 0.033(0.0-18.47) |  |  |
| **STAT5a** |  |  |  |
| **HC** | 0.47(0.0024-57.43) | 0.66 | 0.01 |
| **RA** | 1.65(0.10-31.47) |  |  |
| **HIF1A** |  |  |  |
| **HC** | 0.035(0.0-10.72) | 0.65 | 0.01 |
| **RA** | 0.007(0.0-1.879) |  |  |
| **RORc** |  |  |  |
| **HC** | 0.008(0.0-0.05) | 0.63 | 0.033 |
| **RA** | 0.011(0.0-0.227) |  |  |
| **SMAD2** |  |  |  |
| **HC** | 0.12(0.0001-21.41) | 0.56 | 0.34 |
| **RA** | 0.17(0.001-10.14) |  |  |
| **SMAD4** |  |  |  |
| **HC** | 1.47(0.0-336.1) | 0.56 | 0.35 |
| **RA** | 3.01(0.0-446.5) |  |  |
| **FOXP3** |  |  |  |
| **HC** | 0.01(0.0-0.03) | 0.52 | 0.68 |
| **RA** | 0.011(0.0-0.268) |  |  |

**Table 3S.** Relative expression level of examined genes in OA patients and healthy subjects (HC) based on the area under the ROC - AUC (Area Under Curve) curve.

| **GEN** | **mediana (IQR)** | **AUC** | **p*** |
| --- | --- | --- | --- |
| **FOXP3** |  |  |  |
| **HC** | 0.01(0.0-0.03) | 0.81 | <0.0001 |
| **OA** | 0.02(0.005-0.188) |  |  |
| **RORc** |  |  |  |
| **HC** | 0.008(0.0-0.05) | 0.73 | 0.0015 |
| **OA** | 0.0185(0.0-1.139) |  |  |
| **SMAD2** |  |  |  |
| **HC** | 0.12(0.0001-21.41) | 0.73 | 0.003 |
| **OA** | 0.17(0.001-10.14) |  |  |
| **STAT5a** |  |  |  |
| **HC** | 0.47(0.0024-57.43) | 0.68 | 0.02 |
| **OA** | 1.65(0.10-31.47) |  |  |
| **SMAD4** |  |  |  |
| **HC** | 1.47(0.0-336.1) | 0.63 | 0.08 |
| **OA** | 3.01(0.0-446.5) |  |  |
| **STAT3** |  |  |  |
| **HC** | 0.34(0.0-57,42) | 0.55 | 0.44 |
| **OA** | 2.78(0.0-63.54) |  |  |
| **SOCS1** |  |  |  |
| **HC** | 0.02(0.0-11.53) | 0.53 | 0.65 |
| **OA** | 0.17(0.0-18.32) |  |  |
| **HIF1A** |  |  |  |
| **HC** | 0.035(0.0-10.72) | 0.52 | 0.78 |
| **OA** | 0.007(0.0-1.879) |  |  |
| **SMAD3** |  |  |  |
| **HC** | 0.0014(0.0-3.304) | 0.50 | 0.97 |
| **OA** | 0.033(0.0-18.47) |  |  |

**Table 4S.** Relative expression level of examined genes in RA patients and OA patients based on the area under the ROC - AUC (Area Under Curve) curve.

| **GEN** | **mediana (IQR)** | **AUC** | **p*** |
| --- | --- | --- | --- |
| **FOXP3** |  |  |  |
| **RA** | 0.011(0.0-0.268) | 0.75 | 0.0007 |
| **OA** | 0.02(0.005-0.188) |  |  |
| **SOCS1** |  |  |  |
| **RA** | 0.02(0.0-11.53) | 0.74 | 0.001 |
| **OA** | 0.17(0.0-18.32) |  |  |
| **SMAD3** |  |  |  |
| **RA** | 0.0014(0.0-3.304) | 0.66 | 0.03 |
| **OA** | 0.033(0.0-18.47) |  |  |
| **RORC** |  |  |  |
| **RA** | 0.011(0.0-0.227) | 0.65 | 0.045 |
| **OA** | 0.0185(0.0-1.139) |  |  |
| **HIF1A** |  |  |  |
| **RA** | 0.035(0.0-10.72) | 0.62 | 0.09 |
| **OA** | 0.007(0.0-1.879) |  |  |
| **STAT3** |  |  |  |
| **RA** | 0.34(0.0-57,42) | 0.60 | 0.18 |
| **OA** | 2.78(0.0-63.54) |  |  |
| **SMAD2** |  |  |  |
| **RA** | 0.12(0.0001-21.41) | 0.58 | 0.30 |
| **OA** | 0.17(0.001-10.14) |  |  |
| **SMAD4** |  |  |  |
| ***RA*** | 1.47(0.0-336.1) | 0.56 | 0.40 |
| **OA** | 3.01(0.0-446.5) |  |  |
| **STAT5a** |  |  |  |
| **RA** | 0.47(0.0024-57.43) | 0.56 | 0.40 |
| **OA** | 1.65(0.10-31.47) |  |  |
|  |  |  |  |

**Figure 11S.** Expression of analyzed genes in whole blood in RA on MTX therapy and RA patients on biological treatment.
